# Synthesis, Crystal Structure, and Antibacterial Properties of Silver-Functionalized Low-Dimensional Layered Zirconium Phosphonates

**DOI:** 10.1021/acs.inorgchem.1c03565

**Published:** 2022-01-19

**Authors:** Morena Nocchetti, Anna Donnadio, Eleonora Vischini, Tamara Posati, Cristiano Albonetti, Davide Campoccia, Carla Renata Arciola, Stefano Ravaioli, Valentina Mariani, Lucio Montanaro, Riccardo Vivani

**Affiliations:** † Department of Pharmaceutical Sciences, University of Perugia, Via del Liceo, 1, 06123 Perugia, Italy; ‡ Institute of Organic Synthesis and Photoreactivity, National Research Council, via P. Gobetti 101, 40129 Bologna, Italy; § Consiglio Nazionale delle Ricerche, Istituto per lo Studio dei Materiali Nanostrutturati (CNRISMN), 40129 Bologna, Italy; ∥ Laboratorio di Patologia delle Infezioni Associate all’Impianto, IRCCS Istituto Ortopedico Rizzoli, via di Barbiano 1/10, 40136 Bologna, Italy; ⊥ Department of Experimental, Diagnostic, and Specialty Medicine, University of Bologna, via San Giacomo 14, 40126 Bologna, Italy; # Laboratorio di Immunoreumatologia e Rigenerazione Tissutale, IRCCS Istituto Ortopedico Rizzoli, via di Barbiano, 1/10, 40136 Bologna, Italy

## Abstract

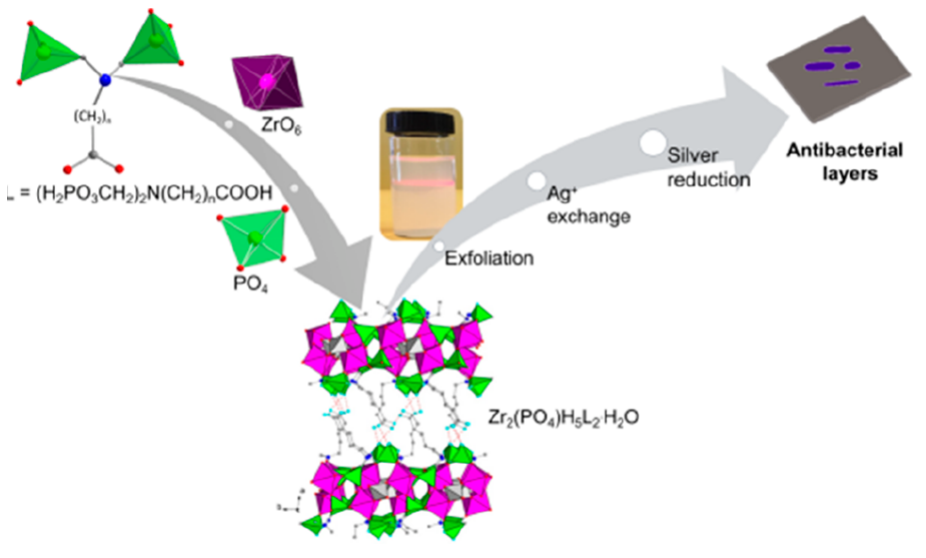

New insoluble layered zirconium phosphate carboxyaminophosphonates (ZPs), with the general formula Zr_2_(PO_4_)H_5_[(O_3_PCH_2_)_2_N(CH_2_)_*n*_COO]_2_·*m*H_2_O (*n* = 3, 4, and 5), have been prepared and characterized. The crystal structure for *n* = 3 and 4 samples was determined *ab initio* from X-ray powder diffraction data. The structure for *n* = 3 was monoclinic in space group *C*2/*c* with the following unit cell parameters: *a* = 34.346(1) Å, *b* = 8.4930(2) Å, *c* = 9.0401(2) Å, and β = 97.15(1)°. The structure for *n* = 4 was triclinic in space group *P*1̅ with the following unit cell parameters: *a* = 17.9803(9) Å, *b* = 8.6066(4) Å, *c* = 9.0478(3) Å, α = 90.466(3)°, β = 94.910(4)°, and γ = 99.552(4)°. The two structures had the same connectivity as Zr phosphate glycine diphosphonate (*n* = 1), as previously reported. By intercalation of short amines, these layered compounds were exfoliated in single lamella or packets of a few lamellae, which formed colloidal dispersions in water. After a thorough characterization, the dispersed lamellae were functionalized with Ag nanoparticles, which were grown *in situ* on the surface of exfoliated lamellae. Finally, their antimicrobial activity was tested on several Gram-positive and Gram-negative bacteria. All of these systems were found to be active against the four pathogens most frequently isolated from orthopedic prosthetic infections and often causative of nosocomial infections. Interestingly, they were found to express powerful inhibitory activity even against bacterial strains exhibiting a relevant profile of antibiotic resistance such as *Staphylococcus aureus* ATCC 700699.

## Abstract 2

Antibacterial single layers supporting AgNPs were prepared by exfoliation and functionalization of two-dimensional crystals of isoreticular zirconium phosphate carboxyaminophosphonates.

## 1 Introduction

Layered materials have been the subject of great interest due to their reactivity that involves many fields of materials chemistry, such as intercalation, ion exchange, catalysis, and photophysical processes. These systems typically consist of the packing of two-dimensional (2D) macrounits with dimensions from tenths to thousands of nanometers in two dimensions and a very narrow thickness that can be <1 nm. Most of the studies of layered materials, including both fundamental and applied studies, have been focused on bulk solids. Applications of these systems cover a wide range of fields such as catalysis, biomaterials, energy, filler of polymeric composites, and water remediation. The performance of 2D materials can be improved, and new and unique properties may be expressed upon their exfoliation in single layers or packets of few layers due to the huge increase in the surface area and the number of easily accessible active sites.^1^ Moreover, “superlattice-like heterostructures” obtained by combining two different types of nanosheets can be synthesized.^2,3^ The nanosheets interact via van der Waals forces and exhibit synergistic effects in their catalytic, chemical, and optical properties.^4−9^ Exfoliated 2D materials, due to their large surface area, can immobilize and stabilize large amounts of metals with antibacterial activity, such as Zn,^10^ Ag,^11,12^ and Cu,^13^ in the form of both cations and metal nanoparticles (NPs). These functionalized nanosheets can be used singly or in combination with other nanosheets (superlattice-like heterostructures) as components of hydrogels, scaffolds for skin, bone, and cartilage tissue repair.^14^

Several 2D materials can serve as a source of monolayers, among them graphene,^15^ transition-metal dichalchogenides (TMDs; MS_2_ where M = Mo, W, or Re),^16^ boron nitride,^17^ phosphorenes [black phosphorus (BP)],^18,19^ MXenes (Ti_3_C_2_ and Ta_4_C_3_),^20^ layered double hydroxides (LDH),^21^ metal–organic frameworks (MOFs),^22^ and zirconium phosphate.^23,24^ The first exfoliation of 2D materials was achieved by a mechanical approach by applying Scotch tape on graphite.^25^ Unfortunately, such a method is not suitable for a large variety of 2D materials, and different synthetic strategies, classified into bottom-up and top-down, have been employed.^26^ Bottom-up approaches, such as chemical vapor deposition, physical vapor deposition, and van der Waals epitaxy techniques, require hard synthetic conditions and are not suitable for a large scale production.^1^ Conversely, top-down strategies, including liquid exfoliation^27,28^ and intercalation followed by exfoliation,^29^ sometimes assisted by physical techniques,^30^ generally require mild conditions and can be scaled. Exfoliation with top-down approaches occurs when the intralayer interactions are overcome; thus, the layers of graphene and TMDs, which interact with van der Waals interactions, are easily separated; densely packed materials such as MOF and LDH have higher packing energies, and their exfoliation is more difficult. Generally, zirconium phosphonates are included in the latter typology of materials due to the high density of organic moieties in the interlayer region. Only a few examples of exfoliation of zirconium phosphonates can be found in the literature. The exfoliation of mixed zirconium 4-sulfophenylphosphonate phenylphosphonate has been achieved in the liquid phase upon intercalation of amino alcohols, tetrabutylammonium hydroxide and triethylamine, under the sonication or by the action of high shear forces.^31^ Recently, our research group has synthesized a zirconium phosphate phosphonate based on glyphosine,^32^ with the formula Zr_2_(PO_4_)H_5_[(O_3_PCH_2_)_2_N(CH_2_)COO]_2_ (hereafter **1**); the presence of acidic -COOH and -POH groups on the surface of the layers enables efficient interactions with basic molecules and cations. Furthermore, the high polarity of the layer surfaces and the reduced level of crowding of organic groups in the interlayer space have allowed the preparation of colloidal dispersions of **1** by liquid-phase exfoliation assisted by the intercalation of short chain alkylamines (as methylamine or propylamine). Metal NPs have been grown and stabilized on the nanosheets of **1** by exchanging the protons with metal cations followed by metal reduction. Highly active PdNPs supported on **1** have shown superior catalytic performance in carbon–carbon bond formation^33,34^ and hydrogenation^35^ reactions. The immobilization of AgNPs or Zn^2+^ ions on exfoliated **1** has allowed the preparation of antimicrobial nanosheets.^36,37^ The aim of this work is the preparation of novel isostructural zirconium phosphate phosphonates (ZPs) based on phosphonic acids, analogous to glyphosine, that can exfoliate under mild conditions. In particular, bis(phosphonomethyl)aminocarboxylic acids with increased carbon chain lengths and phosphoric acid have been used in the ZP synthesis. The structure of ZP samples has been determined *ab initio* from X-ray powder diffraction (XRPD) data, and their reactivity studied. Particular attention has been paid to the preparation of stable and concentrated ZP dispersions via exfoliation in an aqueous solution. These dispersions have been characterized by XRPD, attenuated total reflectance-Fourier transform infrared spectroscopy (ATR-FTIR), transmission electron microscopy (TEM), and atomic force microscopy (AFM). Finally, ZP nanosheets have been shown to have the ability to immobilize AgNPs and to release them in a controlled manner. Moreover, the antimicrobial activity has been tested on five different bacterial reference strains exhibiting powerful inhibitory activity.

## 2 Materials and Methods

### 2.1 Materials

All of the chemicals were purchased from Sigma-Aldrich and used as received without further purification.

### 2.2 Synthesis of *N*,*N*-Bis(phosphonomethyl)aminocarboxylic Acids

The *N*,*N*-bis(phosphonomethyl)aminocarboxylic acids (H_2_O_3_PCH_2_)_2_N(CH_2_)_*n*_COOH [where *n* = 1, 3, 4, and 5 (H_5_L in general)] were prepared as reported previously in ref (38). Briefly, 67 mmol of H_2_N(CH_2_)_*n*_COOH and 11 g of H_3_PO_3_ (133 mmol) were dissolved in 50 mL of 6 M HCl. This mixture was heated to reflux, and 8 g of paraformaldehyde (266 mmol), dispersed in 10 mL of water, were slowly added within 2 h. After the last addition of paraformaldehyde, the solution was refluxed again for 1 h and then the solvent was evaporated. The raw mixture was treated with 2-propanol, yielding a white solid that was filtered under vacuum and dried in an oven at 60 °C.

### 2.3 Synthesis of Zirconium Phosphate Phosphonates

Microcrystalline Zr_2_(PO_4_)H_5_[(O_3_PCH_2_)_2_N(CH_2_)_*n*_COO]_2_·*m*H_2_O compounds (hereafter **1** for *n* = 1 and *m* = 1, **2** for *n* = 3 and *m* = 0.6, **3** for *n* = 4 and *m* = 0.7, and **4** for *n* = 5 and *m* = 1.0; ZP in general) were prepared as reported in ref (32) for **1**. Briefly, 9 mmol of H_5_L was solubilized in 93 mL of a proper solvent [water for **1** and **2**, 1:9 (v/v) propanol/water for **3**, and 1:1 (v/v) propanol/water for **4**], and then 6 mL of 1 M phosphoric acid was added to this solution (H_5_L:phosphoric acid molar ratio of 1.5). Separately, 1.93 g (5.9 mmol) of a zirconium oxychloride octahydrate solution was dissolved in 20.4 mL of 2.9 M hydrofluoric acid (59 mmol; HF:Zr molar ratio of 10). These two solutions were mixed in a 500 mL plastic bottle and placed in an oven at 90 °C. The final P:Zr molar ratio was 4. After 2 h, a white precipitate began to form, and 3 days later, the solid was filtered, washed with deionized water, and dried at 60 °C for 24 h. The solid was conditioned over MgCl_2_ (33% relative humidity) for 1 week. Yields (calculated on the basis of Zr) of 68% for **1**, 67% for **2**, 36% for **3**, and 70% for **4**.

The composition of the anhydrous samples dried at 120 °C has been obtained by inductively coupled plasma-optical emission spectroscopy (ICP) and elemental analysis.

Anal. Calcd (found) for **1**: Zr, 22.8 (22.4); P, 19.4 (18.8); C, 12.0 (11.6); N, 3.5 (3.3); H, 2.1 (2.5). P:Zr molar ratio of 2.36

Anal. Calcd (found) for **2**: Zr, 21.3 (21.2); P, 18.1 (18.2); C, 16.7 (20.8); N, 3.3 (3.9); H, 2.9 (3.8). P:Zr molar ratio of 2.39

Anal. Calcd (found) for **3**: Zr, 20.6 (19.9); P, 17.6 (16.2); C, 19.1 (18.4); N, 3.2 (3.0); H, 3.3 (3.6). P:Zr molar ratio of 2.44

Anal. Calcd (found) for **4**: Zr, 20.0 (19.5); P, 17.0 (16.2); C, 21.1 (20.3); N, 3.1 (3.1); H, 3.6 (3.3). P:Zr molar ratio of 2.42

### 2.4 Delamination of ZP with Amines and Regeneration in a Protonated Form

One gram of ZP was suspended in 100 mL of water, and then 0.1 M *n*-propylamine (PrNH_2_) or methylamine (MeNH_2_) in a Amine:ZP molar ratio of 3:1 was added (37 mL for **1**, 35 mL for **2**, 34 mL for **3**, and 32 mL for **4**) under vigorous magnetic stirring.

The dispersion was then treated with a suitable volume of 1 M HCl (to reach pH <2) to remove the amine and regenerate the solid in its fully protonated form. The resulting solid was separated from the solution and washed with water under vigorous stirring. A gelatinous precipitate was settled by centrifugation at 5000 rpm. Washing was repeated until the samples were free of chloride ions. In the following, these gel samples are labeled **1g–4g**, respectively, and ZPg in general. The gels were suspended in 50 mL of deionized water and then stored in closed containers. To determine the content of anhydrous ZP in the dispersions, a known volume of each dispersion was dried in an oven at 100 °C up to a constant weight. The content of ZP in each dispersion was 18.2 mg/mL for **1g**, 19.5 mg/mL for **2g**, 18.9 mg/mL for **3g**, and 19.1 mg/mL for **4g**.

### 2.5 Preparation of Ag@ZP

Samples containing silver were prepared starting from 12.5 mL of aqueous dispersions of **2g–4g**. In detail, aqueous 0.06 M AgCH_3_COO in a Ag:ZP molar ratio of 0.375 was added dropwise to the ZPg dispersions (1.8 mL for **2g**, 1.7 mL for **3g**, and 1.6 mL for **4g**) under vigorous stirring. The dispersions were left under magnetic stirring for 24 h, and then the materials were collected by centrifugation (15000 rpm for 10 min) and washed twice with water. The recovered samples were equilibrated for 12 h at room temperature in 80 mL of ethanol. Finally, they were recovered by centrifugation, washed twice with water, and dispersed in 12 mL of deionized water. The samples were named **Ag@2**, **Ag@3**, and **Ag@4**, respectively, and Ag@ZP in general. To determine the content of anhydrous Ag@ZP in the dispersions, a known volume of each dispersion was dried in an oven at 100 °C up to a constant weight. The content of Ag@ZP in each dispersion was 19.5 mg/mL for **Ag@2**, 18.5 mg/mL for **Ag@3**, and 18.5 mg/mL for **Ag@4**.

### 2.6 Silver Release Test

Studies of the release of Ag^+^ from Ag@ZP were performed as reported in ref (36). Briefly, 8 mL of a Ag@ZP dispersion was added to 50 mL of minimum essential medium (MEM) cell culture medium at 37 °C. The amount of silver released was determined over 24 h; withdrawals of 2 mL of the acceptor fluid (MEM) were performed, and the same volume of MEM, equilibrated at 37 °C, was immediately replaced. The amount of silver released was detected by ICP after having diluted the taken MEM to 10 mL with 5 M HNO_3_.

Tests were performed in triplicate, and the results were reported as an average and normalized on the basis of the total silver content.

### 2.7 Characterization of Antibacterial Properties

#### 2.7.1 Sample Preparation

The antibacterial activity of Ag@ZP was assayed by an agar diffusion test. A few drops of the dispersions of **Ag@2** (containing 19.5 mg of solid/mL), **Ag@3** (containing 18.5 mg of solid/mL), and **Ag@4** (containing 18.5 mg of solid/mL) were deposited on both sides of filter paper disks with a diameter of 10 mm. The loaded disks were left to dry at room temperature and weighed to estimate the amount of Ag@ZP deposited: 4.6 ± 0.8 mg per disk for **Ag@2**, 3.0 ± 0.5 mg per disk for **Ag@3**, and 2.9 ± 0.4 mg per disk for **Ag@4**. In addition to the negative control consisting of only untreated paper disks, further controls were prepared with paper disks loaded with ZPg dispersions, as previously described.^35^ These additional control disks contained the following amounts of ZPg: 3.5 ± 0.5 mg/disk for **2g**, 4.0 ± 0.9 mg/disk for **3g**, and 3.8 ± 0.6 mg/disk for **4g**.

#### 2.7.2 Bacterial Strains

The microbiological tests were performed on five different bacterial reference strains: *Staphylococcus epidermidis* RP62A (ATCC 35984), *Staphylococcus aureus* ATCC 25923, *S. aureus* ATCC 700699, *Enterococcus faecalis* (ATCC 29212), and *Pseudomonas aeruginosa* ATCC 27853. These reference strains included the four most prevalent etiological agents of implant-related infections in orthopedics.^39^ Moreover, *S. aureus* ATCC 700699, a methicillin-resistant (MRSA) and vancomycin-intermediate (VISA) strain, was adopted to ascertain the antimicrobial potential of ZP@Ag materials even against antibiotic-resistant bacteria.

#### 2.7.3 Agar Diffusion Assay

Fresh cultures of each bacterial strain were prepared from frozen stocks stored at −80 °C in the ISO 9001:2015 certified biorepository of the Research Unit on Implant Infections of the IRCSS Istituto Ortopedico Rizzoli (Bologna, Italy). Bacteria were first plated on tryptic soy agar plates (TSA, catalog no. B19420, MEUS S.r.l., Piove di Sacco, Italy) at 37 °C overnight. Bacterial suspensions were prepared starting from bacterial colonies and adjusted to an optical density of 0.1 at 625 nm (corresponding to a bacterial suspension of ∼10^8^ CFU/mL), using the HP 8453 ultraviolet–visible spectrophotometer (International PBI, Milan, Italy). Subsequently, bacterial suspensions were seeded on Mueller Hinton Agar (MH II Agar, catalog no. B19372, MEUS) plates. Control and Ag@ZP-loaded disks were placed in the center of the seeded agar plates. In addition to negative and ZPg controls, two types of positive controls were prepared by loading paper disks with 50 units of penicillin and 0.05 mg of streptomycin (penicillin-streptomycin, catalog no. ECB 3001D, Euroclone, Pero, Italy) or with 50 μg of gentamicin (gentamicin solution, Sigma-Aldrich, Milan, Italy). Agar plates were cultured for 24 h at 37 °C. Agar diffusion tests were independently repeated three times. The zone of inhibition was measured using the formula (*D* – *d*)/2, where *D* is the diameter of the halo and *d* is the diameter of the disk. Photos were taken of all agar plates.

### 2.8 Analytical Procedures

Zirconium, phosphorus, and silver contents were obtained by ICP using a Varian Liberty Series II instrument working in axial geometry after the mineralization of samples with hydrofluoric acid and nitric acid.

Carbon, nitrogen, and hydrogen contents were determined by elemental analysis using an EA 1108 CHN Fisons instrument.

XRPD patterns for structure determination and Rietveld refinements were collected with Cu Kα radiation on a Bruker D8 Advance diffractometer equipped with a Lynxeye XE-T detector. The long fine focus (LFF) ceramic tube was operated at 40 kV and 40 mA. To minimize the preferential orientations of the microcrystals, the samples were carefully side-loaded onto a zero-background sample holder.

Field emission scanning electron microscopy (FE-SEM) images were collected with a LEO 1525 ZEISS instrument working with an acceleration voltage of 15 kV. The elemental mapping of metals in samples was conducted by using energy-dispersive X-ray spectroscopy (EDS).

Transmission electron microscopy (TEM) analysis was carried out with a JEOL JEM-2010 high-resolution transmission electron microscope, operating at an accelerating voltage of 200 kV. Powders were rapidly diluted in water to avoid aggregation phenomena, supported on copper grids (200 mesh) precoated with a Formvar film, and quickly dried.

Attenuated total reflectance-Fourier transform infrared spectroscopy (ATR-FTIR) measurements were carried out with a Shimadzu IR-8000 spectrophotometer. The spectral range collected was from 400 to 4000 cm^–1^, with a spectral resolution of 4 cm^–1^ acquiring 100 scans.

Ion exchange titrations were performed with a Radiometer automatic titrimeter (TIM900 Titrilab and ABU91 Buret); 0.1 g of solid was suspended under stirring in 10 mL of 0.1 M added salt and titrated by adding 0.1 mL of 0.1 M solution titrant every 60 s.

Thermogravimetric (TG) analysis was performed using a Netzsch STA490C thermoanalyser under a 50 mL min^–1^ air flux with a heating rate of 10 °C min^–1^.

The ζ potential, at 25 °C in aqueous solutions (0.15 mg mL^–1^), was determined by photon correlation spectroscopy (PCS) using a NanoBrook Omni Particle Size Analyzer (Brookhaven Instruments Corp., Holtsville, NY) equipped with a 35 mW red diode laser (nominal 640 nm wavelength).

Topographic images of ZP microcrystals were collected by AFM (SOLVER HV-MFM, NT-MDT Zelenograd, Moscow, Russia) operating in intermittent contact, employing HA_NC cantilevers (NT-MDT, cantilever A, ω_0_ = 235 ± 10 kHz and *k* = 12 ± 2 N m^–1^, ω_0_ = 140 ± 10 kHz and *k* = 3.5 ± 0.7 N m^–1^). Topographic AFM images were processed and morphologically analyzed by Gwyddion software.^40^

### 2.9 Structure Determination and Refinement for **2** and **3**

The crystal structures of **2** and **3** were determined *ab initio* from XRPD data using a simulated annealing (SA) procedure with the help of EXPO2014 software.^41^ This package allowed us first to determine unit cell parameters with NTREOR,^42^ to assign the most probable space group, and finally to apply a SA procedure, using suitable building blocks: one octahedral ZrO_6_, one tetrahedral PO_4_, and one L for **2** (space group *C*2/*c*) and two ZrO_6_, one PO_4_, and two L moieties for **3**, because its asymmetric unit contained one formula unit (space group *P*1̅). The best solutions provided by the SA routine had the same connectivity as **1**; therefore, these models were refined by a Rietveld procedure using the EXPGUI-GSAS package.^43^ During the refinement, soft constraints for bond distances and angles involving C, N, and O atoms were applied, and their statistical weight was decreased as the refinement converged; however, it was not possible to set them to zero, to avoid unrealistic bond distances and angles for the organic part. At the end of the refinement, the shifts in all parameters were smaller than their standard deviations. Nevertheless, estimated standard deviations on bond distances and angles for the organic part are rather high, probably due to some disorder affecting the carboxyalkyl chains. Tables S1–S8 report crystallographic data, refinement details, bond distances, and bond angles, while Figures S1–S4 show the final Rietveld plots and the asymmetric units for **2** and **3**.

## 3 Results and Discussion

### 3.1 Synthesis and Characterization of ZPs

**Scheme 1:**
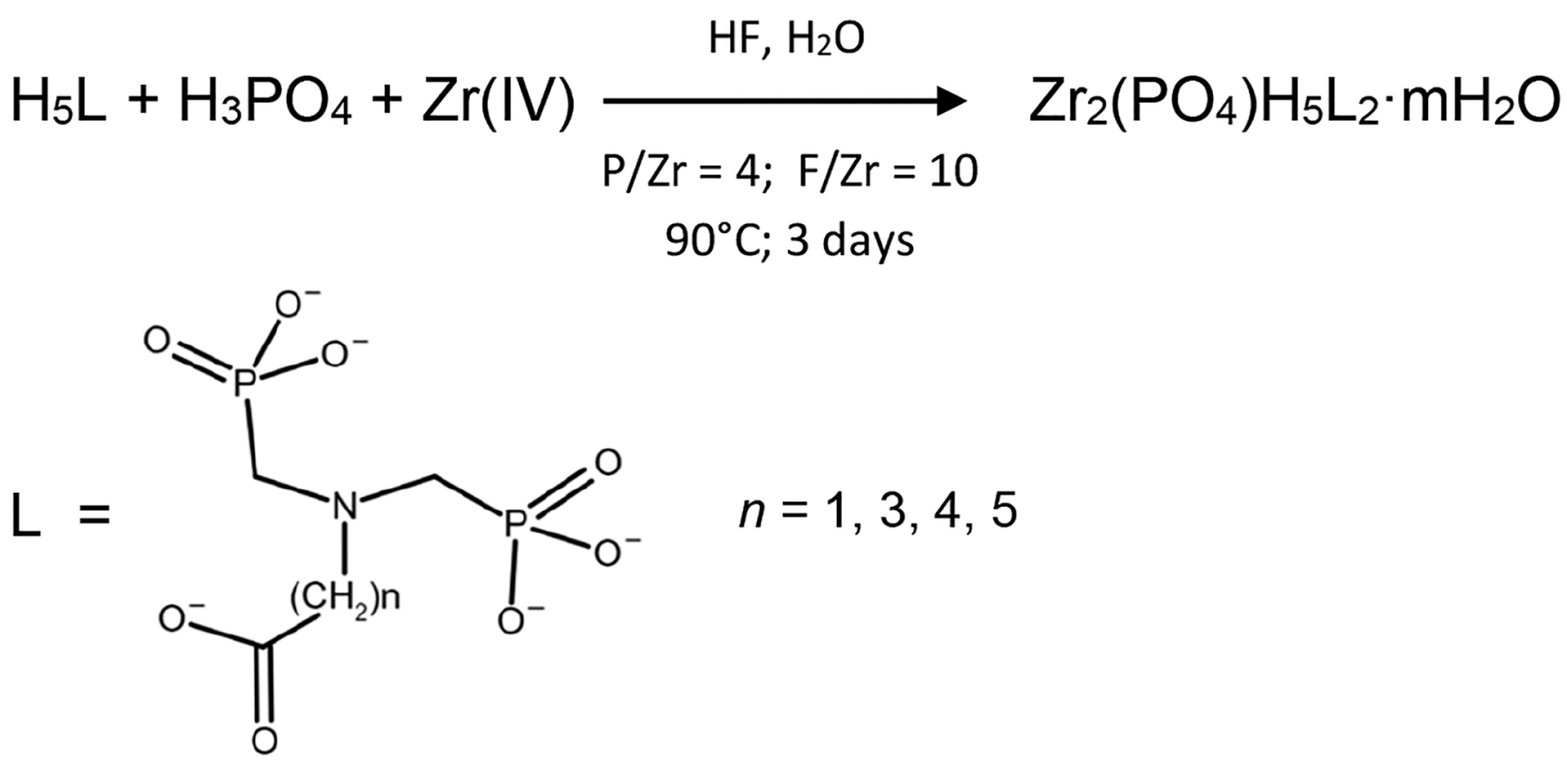
Synthesis of Zr_2_(PO_4_)H_5_L_2_·*m*H_2_O and Molecular Structure of the *N*,*N*-Bis(phosphonomethyl)aminocarboxylate Moiety

Zirconium phosphate bis(phosphonomethyl)aminocarboxylates with different carbon chain lengths were prepared, and their properties were compared with those of the first member, Zr phosphate bis(phosphonomethyl)glycine (**1**).^32^ First, H_5_L building blocks were prepared by means of the Moedritzer–Irani reaction^38^ (Scheme S1) starting from the relative amino acids. The slow decomposition of Zr(IV) fluoro complexes, in the presence of H_5_L and phosphoric acid, produced the formation of microcrystalline Zr phosphate phosphonates (Scheme 1). All of the obtained solids showed P:Zr and L:PO_4_ molar ratios very close to 2.5 and 2, respectively (see Materials and Methods), and a general formula of Zr_2_(PO_4_)H_5_(L)_2_·*m*H_2_O, according to what was previously found for **1**.

**Figure 1:**
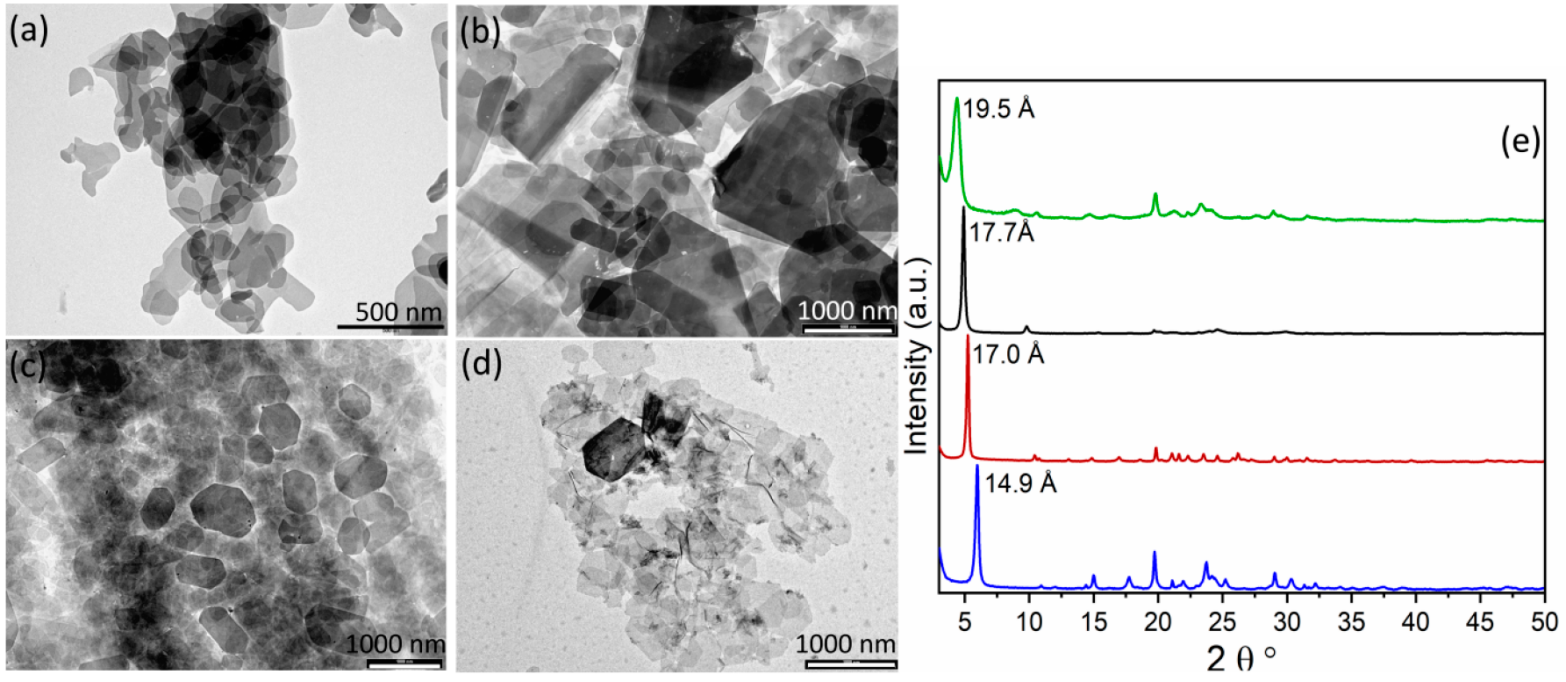
TEM images of (a) **1**, (b) **2**, (c) **3**, and (d) **4**. (e) XRPD patterns of **1** (blue), **2** (red), **3** (black), and **4** (green).

The morphology of the ZP samples has been investigated by TEM, and the collected images are reported in Figure 1a–d. In all of the samples, thin platelet-like sheets are visible with an inhomogeneous size distribution, especially for **2**.

Thermogravimetric data show that all ZPs contain some amount of intercalated water, which starts to be lost at ∼50 °C (Figure S5). For sake of simplicity, the structural studies were performed on anhydrous samples dried at 120 °C.

**Figure 2:**
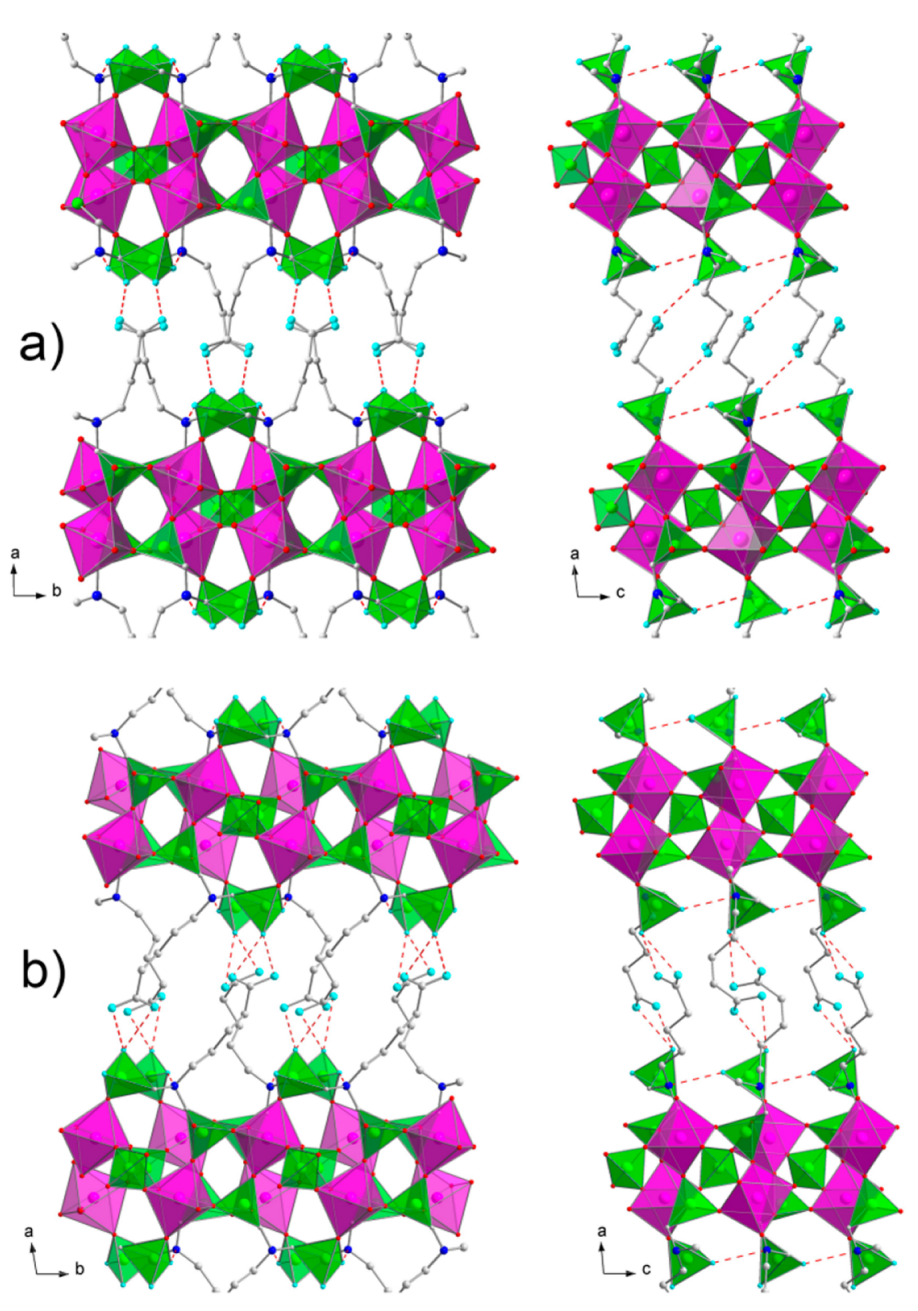
Polyhedral structures of anhydrous (a) **2** and (b) **3**, viewed down the *c* axis on the left and down the *b* axis on the right. ZrO_6_ octahedra are colored purple, and PO_4_ tetrahedra and PO_3_C tetrahedra are colored green. The O terminal atoms are colored cyan. Hydrogen bonds are represented by red dashed lines.

Figure 1e shows the normalized XRPD patterns of new ZPs obtained in comparison with those of **1**. The crystallinity of **2** and **3** allowed us to solve *ab initio* the structure from XRPD data. The structures of anhydrous **2** and **3**, reported in Figure 2, show that they are layered compounds, made of the connection of ZrO_6_ octahedra with PO_4_ phosphate and O_3_PC phosphonate tetrahedra. Their structures differ in symmetry and space group: monoclinic and space group *C*2/*c* for **2** (similar to **1**) and triclinic and space group *P*1̅ for **3**. Despite the different global symmetry, *b* and *c* unit cell parameters, which define the layer texture, and the global connectivity of **2** and **3** are close to those of **1**. For this reason, these ZPs can be considered a rare case, in the chemistry of Zr phosphonates, of isoreticular compounds. Despite numerous efforts, we have not been able to obtain **4** with a sufficiently detailed XRPD pattern to allow us to apply structure determination methods; however, because this compound has exhibited very similar chemical and physical characteristics, we can assume that **4** also has the same connectivity and, therefore, an isoreticular structure.

The first strong peak at low 2*θ* values of the patterns is related to the layer stacking and depends on the length of carboxyalkyl chains. The interlayer distances are 14.9, 17.0, 17.7, and 19.5 Å for **1–4**, respectively.

Each layer of ZP has a complex structure. It is composed of Zr atoms placed in two parallel planes (Figure 2), which are coordinated by tetradentate phosphate groups placed inside the two metal planes, and by the two O_3_PC phosphonate tetrahedra, which are placed outside the two metal planes. These two phosphonate tetrahedra belong to the same amino diphosphonate moiety.

They are not chemically equivalent. One of them is placed further inside the layer and links three different Zr atoms, while the other is placed at the external side of the layer and links only one Zr atom. Of this latter phosphonate, two P–O groups are uncoordinated to Zr; one of them is probably anionic, P–O^–^, because it protonates the nitrogen atom of an adjacent aminophosphonate moiety, as observed in several analogous structures showing similar P–O^–^···H^+^–NR_3_ systems.^32^ This feature, confirmed by ATR-FTIR spectra (see below), gives a zwitterionic character to these solids. Hydrogen bonds are present between P–O^–^ and H^+^–NR_3_. In sample **2**, the P13–O15···N11 distance is 2.86(2) Å, while in **3**, the P13–O15···N11b distance is 2.80(2) Å and the P13b–O15b···N11 distance is 2.79(2) Å. The last P–O group, belonging to this phosphonate group, points toward the interlayer region; for electroneutrality requirements, half of them are P–OH and half are P=O. This P–O group interacts via hydrogen bonds with a carboxylic group coming from an adjacent layer, as already observed in **1**. The aminocarboxylic tails occupy the interlayer region in an interdigitated fashion so that the carboxylate terminal groups can get close to the phosphonate groups of adjacent layers. However, **2** and **3** show different interactions, depending on the relative arrangement of carboxylic and P–O groups.

In **2**, each carboxylic group approaches the P–O with only one oxygen atom and forms one hydrogen bond [P13–O14···O21–C21, 2.75(2) Å]; on the contrary, in **3** the two oxygen atoms of the carboxylate group are approximately equidistant from the P–O and form two hydrogen bonds with similar distances [for one phosphonate, P13–O14···O20–C21 = 2.82(3) Å and P13–O14···O21–C21 = 2.79(2) Å, and for the second phosphonate group in the asymmetric unit, P13b–O14b···O20b–C12b = 2.85(3) Å and P13b–O14b···O21b–C12b = 2.79(2) Å].

This interdigitated arrangement is possible because in this peculiar structure each carboxyalkyl chain has an available cross section of ∼40 Å^2^ (*b* × *c*/2), which is more than twice the cross section of an alkyl chain (18.6 Å^2^).^44^ For this reason, the organic moieties coming from adjacent layers doubly occupy this area and can also assume a relaxed conformation. This environment can easily generate some disorder in the carbon and oxygen positions, which can justify the low resolution obtained for the bond distances and angles of the organic part.

The structure contains five protonated groups per FW, with different acidic strengths, as observed by ion exchange experiments (see the Supporting Information). Two carboxylic groups and one P–OH group are more acidic and can be easily exchanged at pH <9–10, while the protons on the amino groups are not easily exchanged even at higher pH values.

**Figure 3:**
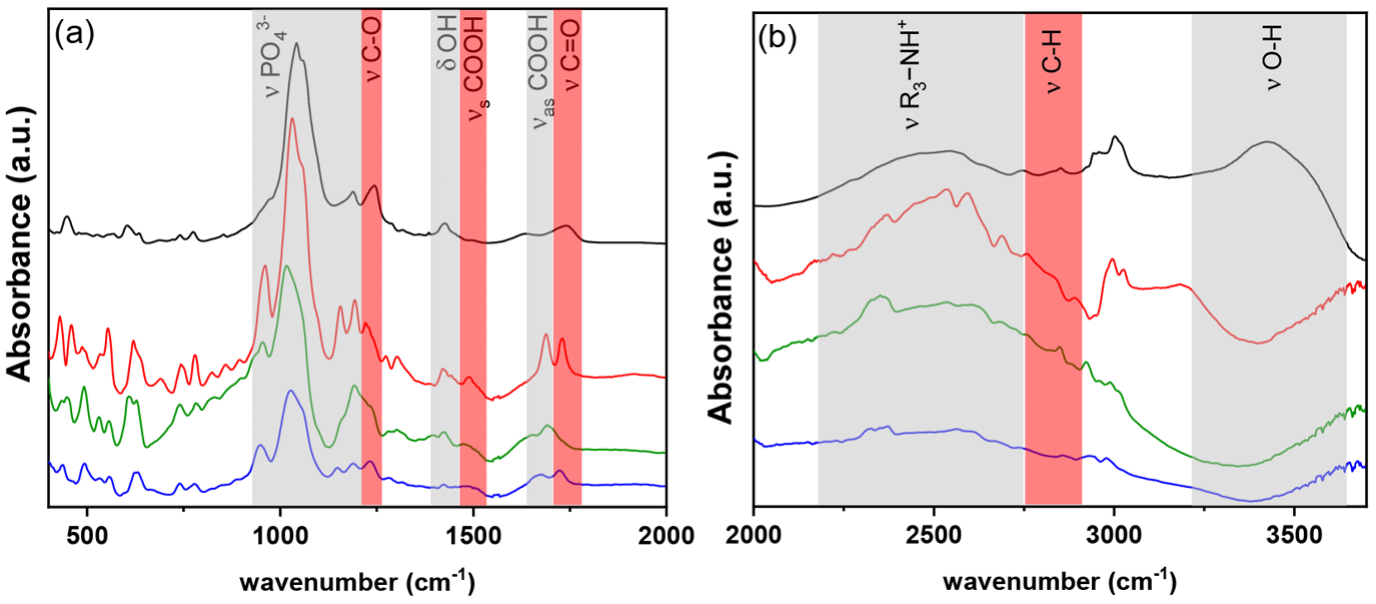
ATR-FTIR spectra of **1** (black), **2** (red), **3** (green), and **4** (blue) in the spectral regions of (a) 400–2000 cm^–1^ and (b) 2000–3700 cm^–1^.

Additional information about the structure of anhydrous ZP samples has been obtained by the ATR-FTIR spectra reported in Figure 3. As discussed in previous work, the ATR-FTIR spectrum of **1** exhibits characteristic absorbance bands at 3000–3500 cm^–1^ assigned to O–H stretching vibrations of the adsorbed water involved in hydrogen bonds. The bands just below 3000 cm^–1^ are due to C–H stretching. The broad band centered at 2600 cm^–1^ is typical of an R_3_N–H^+^ group, evidencing the presence of protonated amino groups and confirming the hypothesis presented above. The 1740 cm^–1^ signal is assigned to the stretching of noncoordinated C=O bonds. The bands at 1424 and 1240 cm^–1^ are due to O–H bending^32^ and C–O stretching, respectively, confirming the presence of COOH groups. The presence of strong absorptions at 940–1200 cm^–1^ is characteristic of P–O stretching motions. In particular, the band at ∼960 cm^–1^ (973 cm^–1^ for **1**, 965 cm^–1^ for **2**, 953 cm^–1^ for **3**, and 949 cm^–1^ for **4**) can be ascribed to PO_4_^3–^ symmetric stretching vibrations ν_1_, and the intense bands in the range of 1020–1150 cm^–1^ are attributed to the HPO_4_^2–^ and PO_4_^3–^ antisymmetric stretching vibrations ν_3_.^45^ The band at 1200 cm^–1^ can be assigned to the P=O stretch. The bands in the region below 900 cm^–1^ can be assigned to various bending modes and Zr–O stretching. The ATR-FTIR spectra of the other solids with longer alkyl chains show some differences. In the spectral range of 500–2000 cm^–1^ (Figure 3a), the main difference can be ascribed to the stretching of the carboxyl group. In **2** and **4**, in addition to ν_C=O_, two bands at 1688 and 1489 cm^–1^ appear and can be ascribed to antisymmetric and symmetric stretching of COOH, respectively, involved in an H-bond network. The network of hydrogen bonds is quite visible in the polyhedral structure of **2** reported in Figure 2 and Figure S2.

In **3**, ν_C=O_ disappears and only the bands associated with the COOH group involved in an H-bond network are detected at 1692 and 1480 cm^–1^. Very likely, the orientation of the COOH in the interlayer region allows all of them to form H-bonds.

In the spectral range of 2000–4000 cm^–1^, the lack of O–H stretching in **2–4** suggests that the increase in the alkyl chain makes the interlayer region more hydrophobic, hindering the rehydration of the sample during the measurement (sample preparation and data acquisition). In **1**, the O–H stretching is detected due to the adsorption of water during the measurement owing to the hydrophilic nature of its lamellae.

### 3.2 Characterization of Colloidal Dispersions of ZPs

The concomitant presence of PO^–^, NH^+^, and COOH groups makes the surface of layers very polar and reactive, allowing efficient interactions with cations or polar molecules. The ion exchange properties of solids were investigated by titrating ZPs with KOH (Figure S6), MeNH_2_, and PrNH_2_ (Figure S7). In general, these experiments highlight the ability of the ZP to exchange hydrogens with cations and to intercalate short alkylamines by an acid–base reaction between the protogenic groups on the layer and the added amine. The titration curves of the ZPs with amines show the typical trend of exfoliated 2D materials in which the active sites involved in the titration are located on the surface of the layer (a deeper discussion of the titration curves is reported in the Supporting Information). Thereby, as already observed for **1**, exfoliation of the solids and colloidal dispersions can be achieved by intercalation of short alkylamines in an aqueous solution.

**Figure 4:**
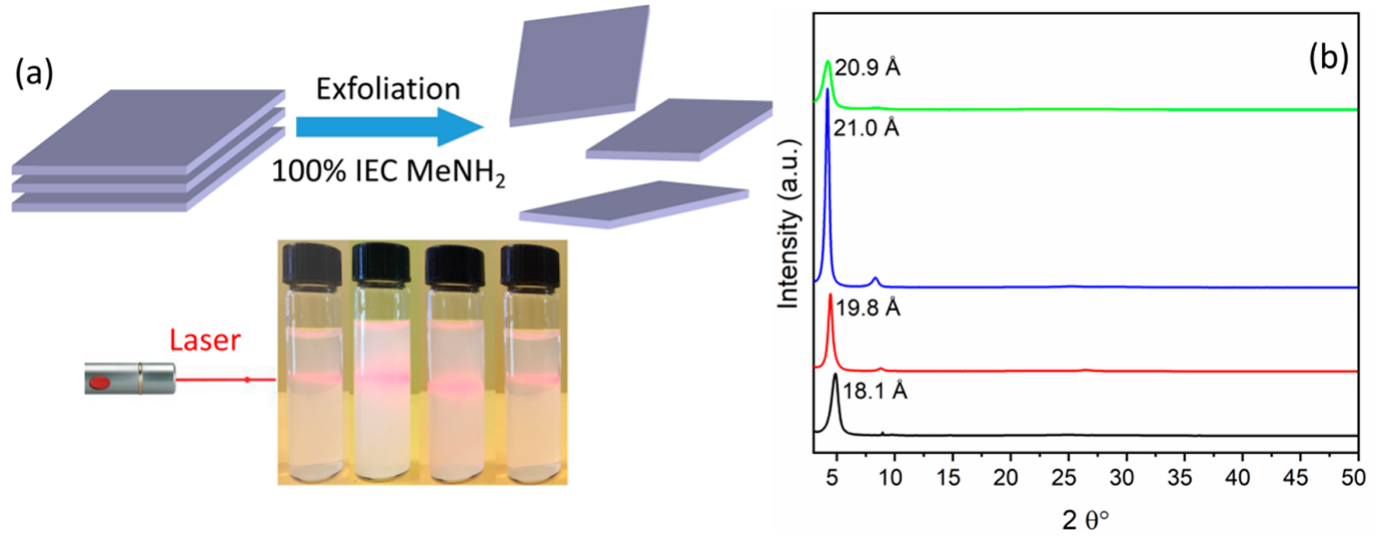
(a) Schematic illustration of ZPs exfoliation by using methylamine and photos of **1–4** (from left to right, respectively), all showing the Tyndall effect. (b) XRPD patterns of air-dried exfoliated **1** (black), **2** (red), **3** (blue), and **4** (green).

A scheme of the exfoliation process by methylamine and images of the obtained exfoliated ZPs are shown in Figure 4a. The stacked ZP layers were separated by the addition of methylamine, which accepts a proton forming methylammonium cations. The latter act as layer balancing cations and interact with the surfaces. The surface polar groups favor the entry of water molecules between the layers, which complete their separation. Very likely, the ability of the ZPs to exfoliate is connected to its unusually large free area associated with each interlayer organic group, which does not allow their closest contacts, thereby reducing the contribution of cooperative van der Waals interlayer interactions to the packing energy. As a result, the introduction of small guest species between the layers by intercalation reactions can easily flake the crystal in single sheets or packets of a few sheets. The potential of these solids to give colloidal dispersions of sheets with a known structure represents an important factor because it allows the use of these materials to immobilize sterically hampered species and, at the same time, to ensure a wide contact with the solution. The colloidal dispersions were drop-cast and air-dried overnight on a glass backing to form a coating of ZP sheets, which was analyzed by XRPD (Figure 4b). Due to the presence of highly oriented lamellae, these patterns are essentially characterized only by the first strong basal peak. The *d* value of these peaks is significantly larger than that of the parent compounds, indicating the presence of the alkylammonium ions and water in the interlayer region.

**Figure 5:**
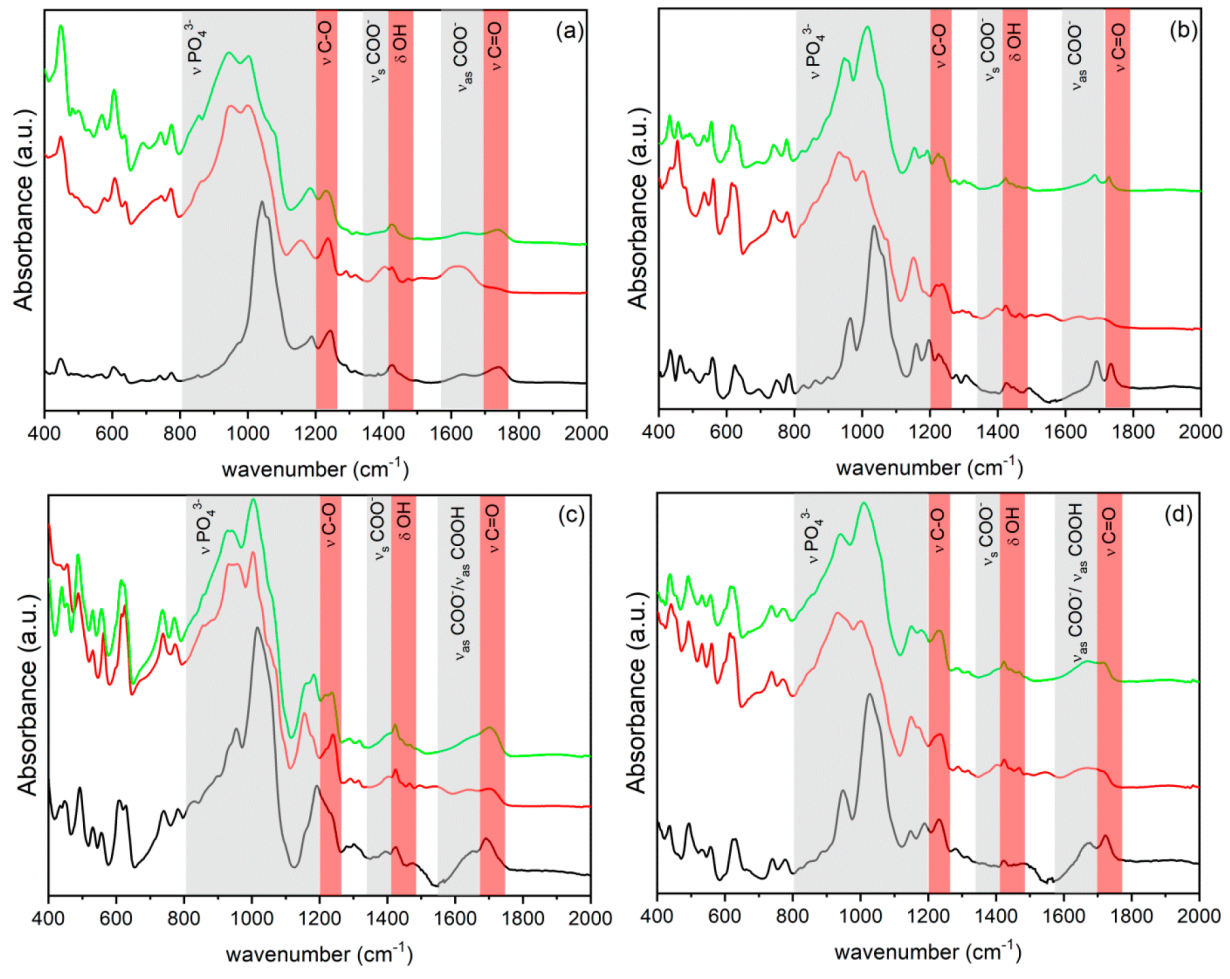
Normalized ATR-FTIR spectra in the spectral region of 400–2000 cm^–1^ of (a) **1**, (b) **2**, (c) **3**, and (d) **4** as synthesized (black), after exfoliation (red), and regenerated with HCl (green).

The ATR-FTIR spectra of the exfoliated ZPs are shown in Figure 5. These spectra are significantly different from those of the starting materials in the spectral range of 400–2000 cm^–1^. The bands that can be ascribed to the C=O and COOH stretching involved in hydrogen bonds are replaced by the antisymmetric and symmetric stretching of the COO^–^ group at 1620 and 1400 cm^–1^, respectively. Moreover, the band at 1620 cm^–1^ is very broad, suggesting the contribution of more than one vibrational mode; the overlapping of the N–H bending of the NH_3_^+^ group with ν_as_ of the COO^–^ group is possible. These findings suggest the protonation of the amine at least by the carboxyl groups and then interaction between −COO^–^ and MeNH_3_^+^. Moreover, the vibration bands of phosphate groups are shifted toward lower wavenumbers; this is an indication of the different interaction of the phosphonic groups that originates from the exfoliation of the solid. The presence of bands in the range of 1000–850 cm^–1^ can be attributed to P–O(H) stretching.^46,47^

The colloidal dispersions of all samples were treated with HCl to completely remove the amine and to obtain dispersions of ZP sheets in their protonated form. ATR-FTIR spectra of ZPg (Figure 5) indicate that in all of the samples the carboxyl groups restore the configuration of the pristine ZPs. The vibrational modes of the phosphates remain unchanged in the **1g** and **3g** samples, suggesting that in these materials the acidic treatments do not provoke a reorganization of the layers, while in the **2g** and **4g** samples, the vibration bands of the phosphate are more similar to those of the pristine **2** and **4** samples. In **4**, a partial restacking of the layers may be expected driven by the chain–chain interaction.

**Figure 6:**
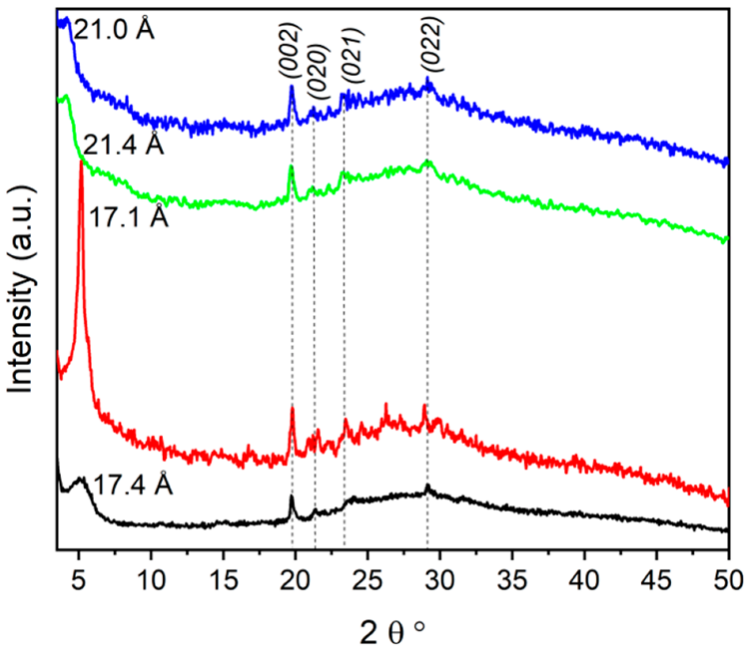
XRPD of **1g** (black), **2g** (red), **3g** (green), and **4g** (blue).

The XRPD patterns of ZPg, reported in Figure 6, deserve a separate discussion. Samples of **1g** and **3g** show a weaker and broader first reflection, and their 2*θ* values were lower than those of **1** and **3**. The corresponding interlayer distances, compared to those of the pristine samples, resulted increases of 2.5 and 3.7 Å for **1g** and **3g**, respectively, indicating that when the exfoliated solids undergo a partial restacking of the layers induced by centrifugation to recover the gel, they tend to incorporate a large amount of hydration water. Very likely, the interactions among the layers in **1g** and **3g** are still very weak as can be deduced by ATR-FTIR spectra. On the contrary, **2g** shows a sharper first reflection than the other ZPg and has the same interlayer distance as **2** while **4g** shows a small increase in interlayer distance, of ∼1.5 Å, compared with that of **4**. These observations can indicate that **2g** and **4g** are more likely to restore the initial interaction as also suggested by ATR-FTIR. In general, the patterns of the ZPg show some reflections at similar 2*θ* values, which can be termed (0*kl*) crystallographic planes having no components along the *a* axis, and therefore only depending on the internal structure of the layers. This observation can indicate that the exfoliation and regeneration processes do not cause changes in the structure of the single layers. After these samples had been completely dried at 80 °C, the formation of the original ZP phase, although less crystalline, was observed. From ICP and elemental analysis data for the ZPg, it was possible to exclude the presence of residual amines (see Table S10) after the regeneration process.

Table 1 shows the *ζ* potentials of the aqueous dispersions of **1g–4g** (pH 6.5), measured to determine the surface charge of the particles. In all cases, these measurements, which provided the average charge of the suspended particles, gave highly negative values, which ranged from −41.60 mV for **3** to −51.40 mV for **1**.^36^ These values of *ζ* potential indicated that the particles of **1g–4g** can also be functionalized with metal ions.

**Table 1:** *ζ* Potentials of ZPg

sample	ζ potential (mV)
**1g**	–51.40 ± 2.72
**2g**	–44.90 ± 0.97
**3g**	–41.60 ± 1.44
**4g**	–46.60 ± 2.38

**Figure 7:**
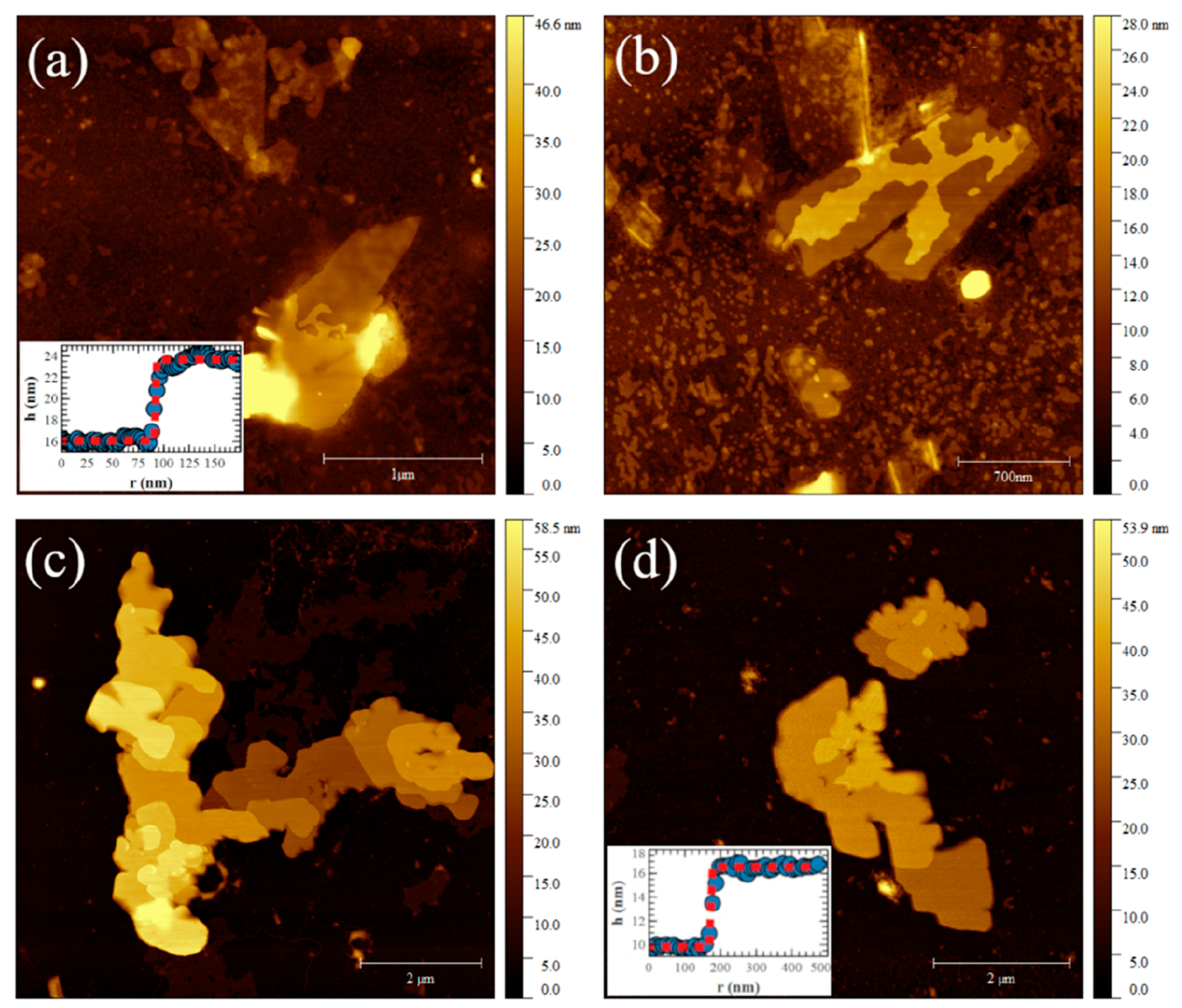
Topographic AFM images of single exfoliated microcrystals of (a) **1**, (b) **2**, (c) **3**, and (d) **4** deposited on Si/SiO_2_ substrates. False color bars highlight stronger and weaker features in white and black, respectively. A multiple-layer step morphology characterizes microcrystal surfaces (see topographic profiles in the insets of panels a and d).

Topographic imaging was performed on samples thus prepared. (i) The dispersions of exfoliated ZPs were diluted 100-fold. (ii) Diluted solutions were deposited on Si/SiO_2_ substrates by the drop-casting technique. (iii) Samples were dried overnight under a laminar hood flow. This experimental procedure reduces surface aggregates, leaving some exfoliated ZP microcrystals homogeneously dispersed on the Si/SiO_2_ surface (see Figure 7).

As shown in Figure 7, the exfoliation process described above produces successfully exfoliated ZP microcrystals. They have both micrometric sizes and shapes comparable to those observed in TEM measurements, together with a crystal surface characterized by a multiple-layer step morphology. Step sizes are randomly distributed; i.e., they are composed of one to three or more ZP single layers (see insets of panels a and d of Figure 7 in which topographic steps composed of five and four layers are shown). The average thickness for each ZP single layer has been statistically calculated by analyzing the height distribution of each microcrystal^48,49^ where small fragments of layers produced by exfoliation and deposited on substrates determine the prevalent height distribution peaks. On average, single-layer thicknesses are ∼15 Å for **1**, ∼18 Å for **2**, ∼19 Å for **3**, and ∼22 Å for **4**. These values are in good agreement with XRPD measurements.

### 3.3 Preparation of Ag@ZP

Sample **1g** revealed a suitable exfoliated support to anchor and stabilize AgNPs, and it showed very good antimicrobial activity.^36,37^ Here the antimicrobial properties of AgNPs grown on **2g–4g** were investigated.

**Figure 8:**
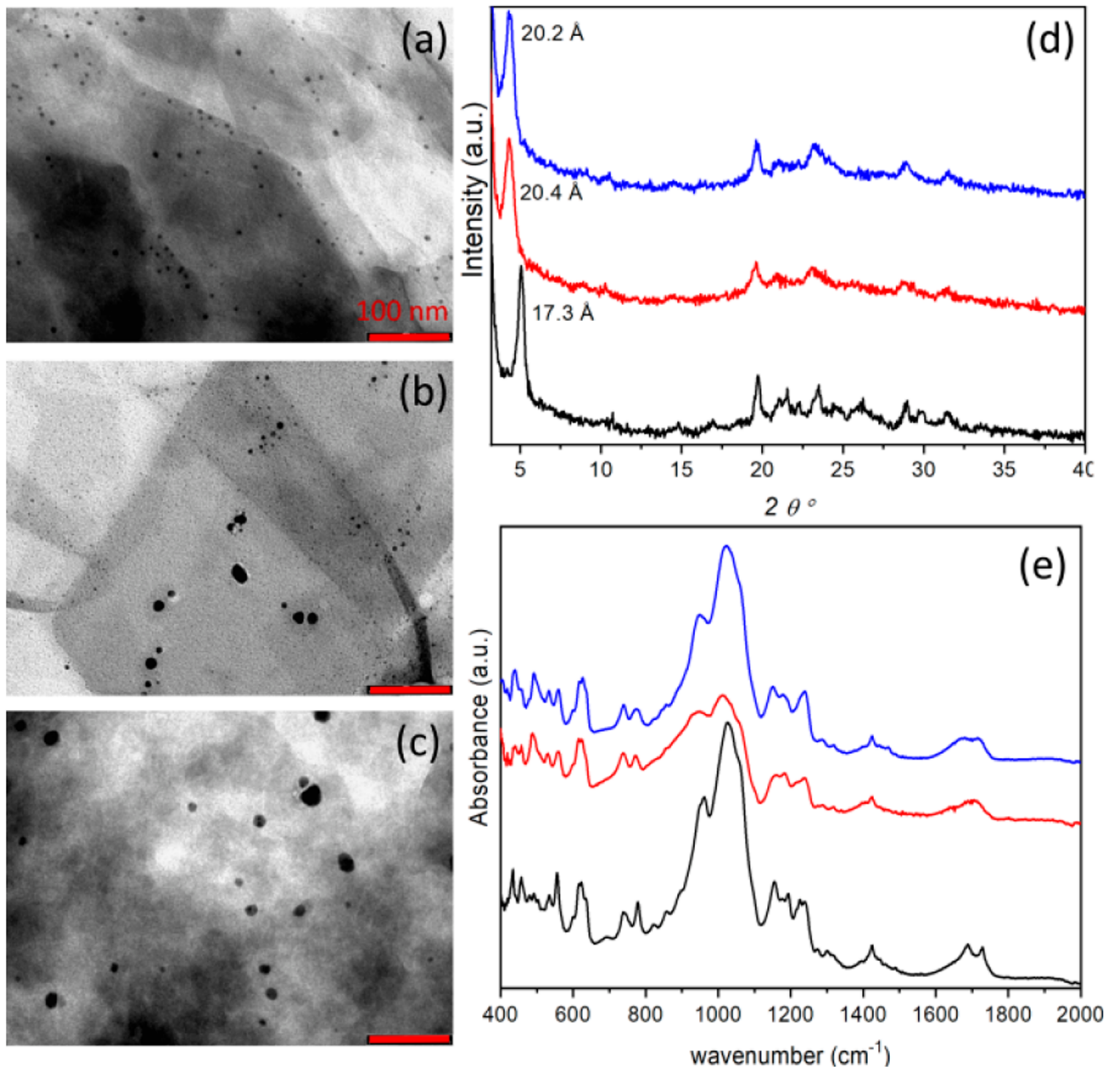
TEM images of (a) **Ag@2**, (b) **Ag@3**, and (c) **Ag@4**. The bar corresponds to 100 nm. (d) XRPD and (e) ATR-FTIR of dried **Ag@2** (black), **Ag@3** (red), and **Ag@4** (blue).

Ag@ZP samples were prepared in two steps that involve Ag^+^/H^+^ exchange on the ZPg followed by the silver reduction in ethanol.^50^Table 2 reports the silver loadings and the AgNP dimensions of Ag@ZP. TEM images of Ag@ZP (Figure 8a–c) show the formation of AgNPs. In **Ag@2**, a population of AgNPs with a diameter of ∼5 nm is present on the layers. **Ag@3** is characterized by two populations of AgNPs of 5 and 15 nm. **Ag@4** presents AgNPs with an average diameter of 20 nm. We note that the longer the alkyl chain, the larger the AgNP dimensions, with **Ag@3** showing a bimodal size distribution; these findings suggest that the environment in which the AgNPs grew was different and very likely more confined in **2** than in **3** and **4**. XRPD and ATR-FTIR data of dried **Ag@2** and **Ag@4**, shown in panels d and e, respectively, of Figure 8, are close to those of the pristine materials, confirming the tendency of the layers to restack regardless of silver immobilization. Conversely, XRPD and ATR-FTIR data of dried **Ag@3** match those of the **3g** sample, attesting to the limited restacking of the layers, as observed in the ATR-FTIR of ZPg of Figure 5.

**Table 2:** Weight Percentages of Silver in the Ag@ZP Samples and AgNP Dimensions Measured by TEM

sample	Ag [% (w/w)]^a^	AgNPs diameter (nm) ± standard deviation^b^
**Ag@2**	2.1	5.2 ± 2
**Ag@3**	4.5	3.5 ± 2
		15 ± 5
**Ag@4**	1.4	20 ± 6

a Data obtained by ICP analyses of the dissolved dried samples.

b Average diameters refer to two different populations of AgNPs observed on TEM images; the values were calculated by measuring at least 100 NPs taken on several images.

**Figure 9:**
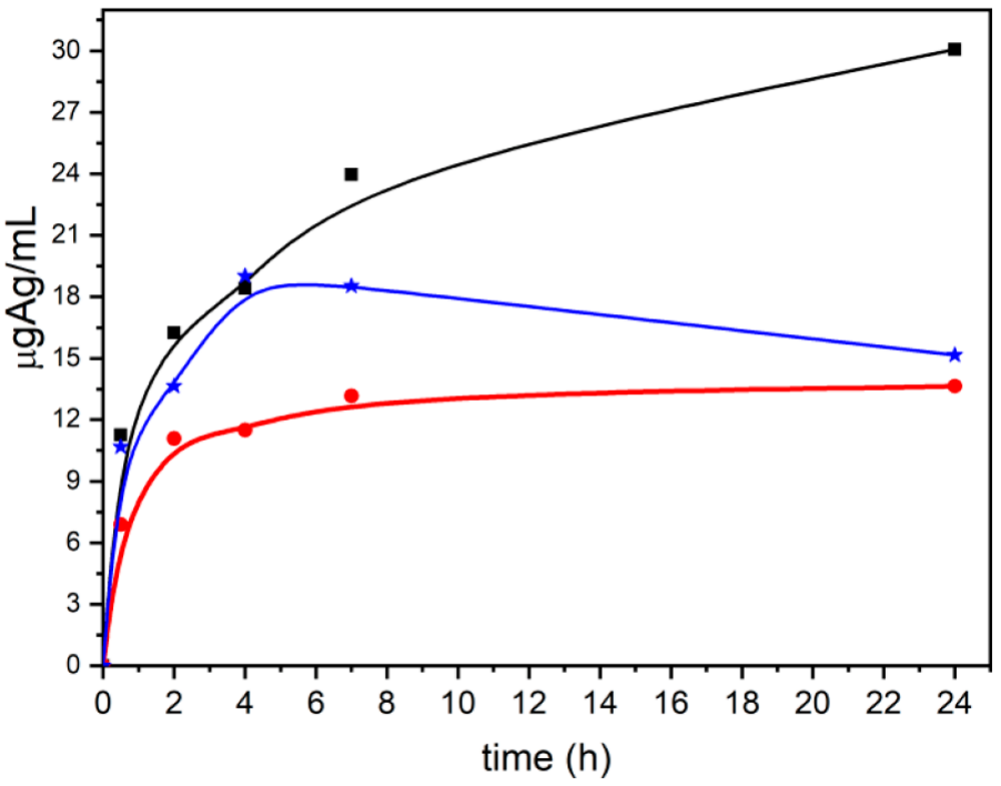
Silver release curves of **Ag@2** (black), **Ag@3** (red), and **Ag@4** (blue) in MEM cell culture medium at 37 °C.

Figure 9 shows the release of silver from Ag@ZP in MEM cell culture medium. The release of **Ag@3** and **Ag@4** is characterized by an initial burst effect, followed by a plateau: **Ag@3** in 4 h and **Ag@4** in 7 h. The percentage of silver released for both of them was ∼40% of the total silver loaded in the samples. Instead, **Ag@2** shows a different behavior; the amount of silver released increases throughout the entire experiment, reaching 47% of the total silver loaded in the sample.

### 3.4 Antibacterial Properties of Ag@ZP

The disk diffusion assay demonstrated broad-spectrum activity for all Ag@ZP materials. As one can see in Figure S8, **Ag@2**, **Ag@3**, and **Ag@4** were found to possess similar antibacterial properties, producing comparable zones of inhibition on the same tested strain. Conversely, the negative controls and the disks loaded with **2g**, **3g**, and **4g** never produced a zone of inhibition around the disks. Overall, staphylococci such as *S. aureus* and *S. epidermidis* appeared to be highly susceptible to the antibacterial activity of all Ag@ZP materials. On the contrary, the growth of *E. faecalis* was inhibited to a lesser extent and agar plates challenged with **Ag@2**, **Ag@3**, and **Ag@4** exhibited small inhibition halos.

**Figure 10:**
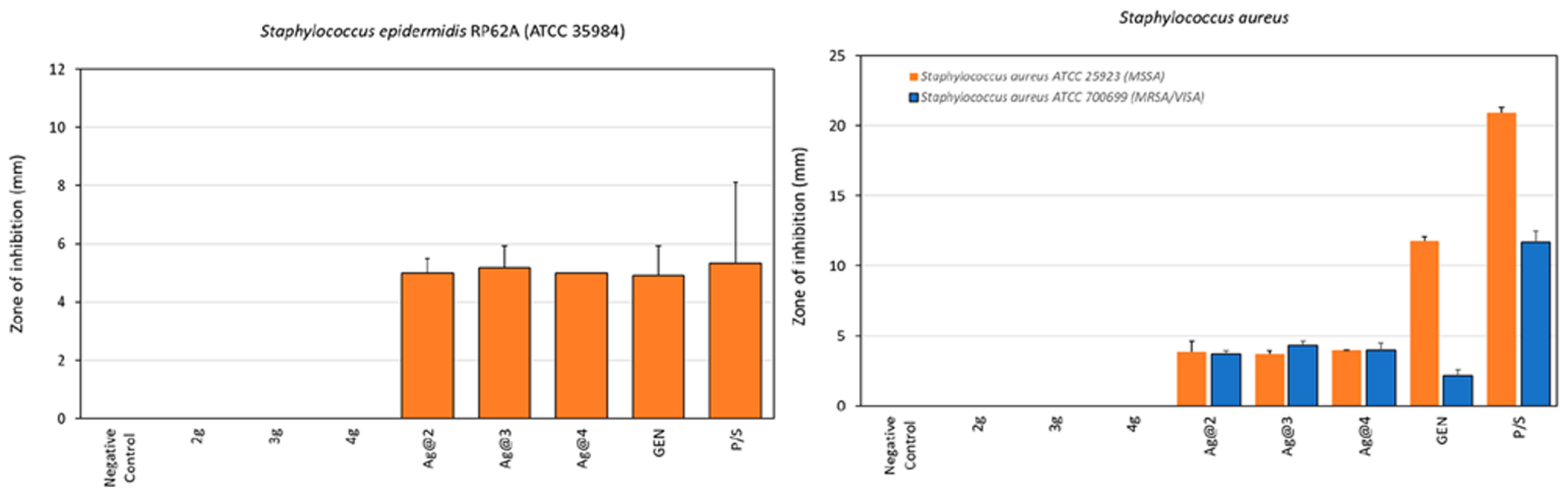
Mean zones of inhibition ± the standard deviation for the different treatments tested on the *S. epidermidis* RP62A reference strain (left) and on the two *S. aureus* reference strains (right). Apart from the positive controls, inhibition halos were observed only in the case of Ag@ZP materials. Interestingly, in *S. aureus* ATCC 700699, the test materials containing Ag were found to produce inhibition similar to that in the methicillin-sensitive strain ATCC25923. On the contrary, both positive controls exhibited a marked reduction in the inhibition halos when tested on the MRSA/VISA strain. Legend: GEN, positive control consisting of gentamicin (50 μg); P/S, positive control consisting of penicillin (50 units) and streptomycin (0.05 mg).

Figure 10 and Table S11 report the size of the inhibition halos observed when Ag@ZP materials were tested on *S. epidermidis* RP62A, a biofilm-producing, methicillin-resistant clinical strain isolated from an intravascular catheter-associated sepsis.^51^ For **Ag@2**, **Ag@3**, and **Ag@4**, the zones of inhibition were approximately 5 mm in size, suggesting a powerful antibacterial activity for all three test Ag@ZP substances.

The measurements of inhibition halos observed when Ag@ZP materials were tested on *S. aureus* were in the range of 3.7–4.0 and 3.7–4.3 mm for the MSSA ATCC 25922 and MRSA/VISA ATCC700699 reference strains, respectively (Figure 10 and Table S12). These results suggest that **Ag@2**, **Ag@3**, and **Ag@4** were equally active on antibiotic-susceptible and antibiotic-resistant *S. aureus* strains. In *S. epidermidis* as well as in both *S. aureus* strains, no significant difference in antimicrobial activity was observed when comparing the effects of **Ag@2**, **Ag@3**, and **Ag@4**.

**Figure 11:**
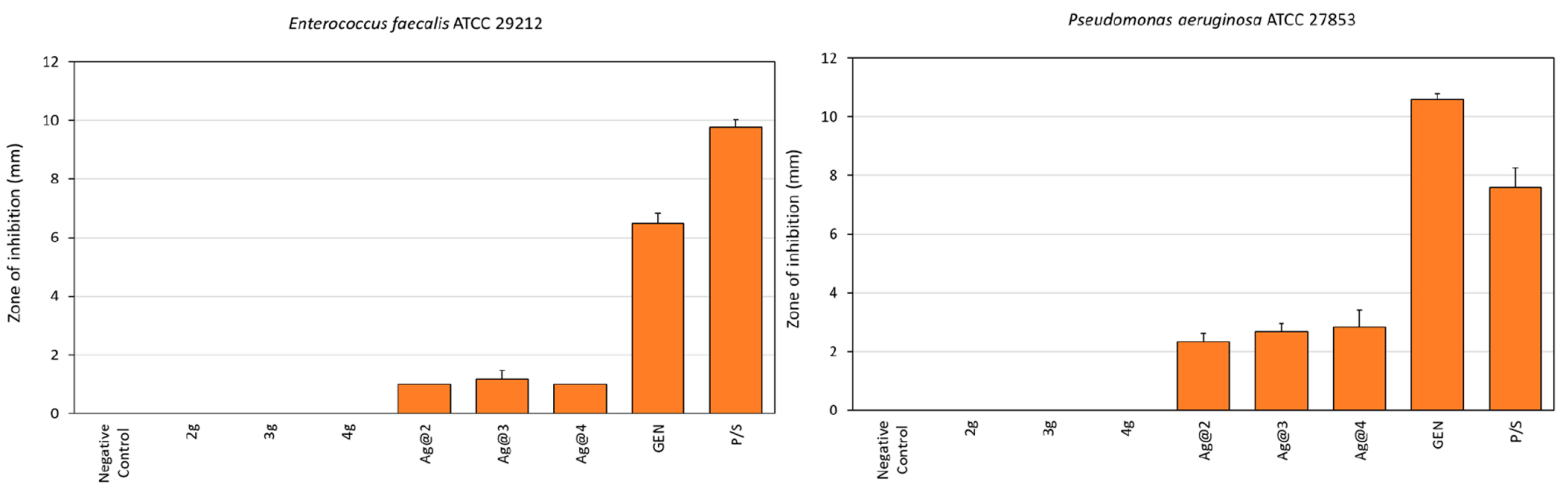
Mean zones of inhibition ± the standard deviation for the different treatments tested on the *E. faecalis* ATCC29212 (left) and *P. aeruginosa* ATCC 27853 (right) reference strains. Apart from the positive controls, inhibition halos were observed only in the case of Ag@ZP materials. **Ag@2**, **Ag@3**, and **Ag@4** produced similar zones of inhibition in both *E. faecalis* and *P. aeruginosa*. However, *E. faecalis* was the species with the lowest susceptibility to the test substances. Legend: GEN, positive control consisting of gentamicin (50 μg); P/S, positive control consisting of penicillin (50 units) and streptomycin (0.05 mg).

The inhibition halos that were produced by disks loaded with Ag@ZP materials on plates seeded with *E. faecalis* ATCC 29212 were much smaller, in the range of 1–1.2 mm (Figure 11 and Table S11). Thus, although capable of inhibiting the growth of *E. faecalis* and endowed with antibacterial properties, **Ag@2**, **Ag@3**, and **Ag@4** appeared to be less effective than when tested on staphylococci.

As far as *P. aeruginosa* is concerned, the three different Ag@ZP materials exhibited similar inhibition zones, ranging from 2.3 mm measured for **Ag@2** to 2.8 mm for **Ag@4** (Figure 11 and Table S13). As in the case of the tests performed on the other bacterial strains, the slight differences observed among the three different Ag@ZP materials were subjected to analysis of variance followed by a Tukey/Kramer test (StatView version 5.0.1, Sas Institute Inc., Cary, NC) and never found to be statistically significant. On the basis of the measurements of the zones of inhibition, the susceptibility to **Ag@2**, **Ag@3**, and **Ag@4** decreased in the following order: *S. epidermidis* > *S. aureus* MSSA = *S. aureus* MRSA > *P. aeruginosa* > *E. faecalis*.

## 4 Conclusions

Zirconium phosphate carboxyaminophosphonates (ZPs) have been prepared with bis(phosphonomethyl)aminocarboxylic acids with different alkyl chain lengths. Except for the caproic derivative, their crystal structure was determined, and although bond distances and angles for the organic part were obtained with rather low resolution, XRPD could provide us with enough information to enable an accurate description of inorganic sheets and elucidation of heavy atom coordination, global connectivity, and weak bond interactions. These compounds are layered and became isostructural with the glycine derivative; only an increase in the interlayer distance was detected, in agreement with the length of the alkyl chain. More interestingly, the solids showed a strong tendency to exfoliate upon ion exchange with methylamine or propylamine, giving the possibility of having a substrate with a larger surface area to immobilize appropriate functional metal ions with anti-infective activity. The XRPD and ATR-FTIR data showed that the exfoliation process before, and regeneration with a strong acid after, did not affect the crystal structure, that was preserved. The highly negative *ζ* potentials indicated that the particles were highly negatively charged and could promote the coordination of metal ions. In particular, by an ion exchange process, silver ions were immobilized onto the nanosheets of ZrPs. We examined metal binding by ICP, ATR-FTIR, and XRPD analysis as well as release studies. **Ag@2**, **Ag@3**, and **Ag@4** demonstrated comparable broad-spectrum antibacterial properties, independent of the length of their alkyl chain. All of them were found to be active against the four pathogens most frequently isolated from orthopedic prosthetic infections and often the cause of nosocomial infections. Interestingly, they were found to exhibit powerful inhibitory activity even against bacterial strains exhibiting a relevant profile of antibiotic resistance such as *S. aureus* ATCC 700699, which not only is resistant to methicillin but also possesses intermediate resistance to vancomycin.

Our preliminary results suggested that these novel amino-carboxyl phosphonates could be potentially used as a highly effective material to support bioactive metal ions or functionalized filler of polymers for biomedical applications.

## Supporting Information Available

Crystallographic data, refinement details, bond distances and angles for **2** and **3** (Tables S1–S8); final Rietveld plots and asymmetric units for **2** and **3** (Figures S1–S4); scheme of the Moedritzer–Irani reaction (Scheme S1); thermal behavior of ZP (discussion, Table S9, and Figure S5); study of ZP reactivity [titration curves with KOH (Figure S6) and titrations with MeNH_2_ and PrNH_2_ (Figure S7)]; characterization of colloidal dispersions of ZP [Zr, P, C, and N contents of the regenerated solids (Table S10)]; and antibacterial properties of Ag@ZP [representative plates of the agar diffusion assay (Figure S8) and zones of inhibition of different Ag@ZP on the tested bacteria (Tables S11–S13)] (PDF).
