# Supplementary material for: Synthesis, Crystal Structure, and Antibacterial Properties of Silver-Functionalized Low-Dimensional Layered Zirconium Phosphonates
Source: Inorg Chem. 2022 Jan 19;61(4):2251–64. doi: 10.1021/acs.inorgchem.1c03565 (PMC9946290; doi:10.1021/acs.inorgchem.1c03565)
Supplement: Supplementary file 1 — ic1c03565_si_001.pdf [file ic1c03565_si_001.pdf]

SYNTHESIS, CRYSTAL STRUCTURE AND ANTIBACTERIAL PROPERTIES OF SILVER FUNCTIONALIZED LOW DIMENSIONAL LAYERED ZIRCONIUM PHOSPHONATES

Morena Nocchetti[1]*, Anna Donnadio[1]*, Eleonora Vischini[1], Tamara Posati[2], Cristiano Albonetti[3], Davide Campoccia[4], Carla Renata Arciola[4,5,6], Stefano Ravaioli[4], Valentina Mariani[4], Lucio Montanaro[4,5], Riccardo Vivani[1]*

[1]Department of Pharmaceutical Sciences, University of Perugia, Via del Liceo, 1, 06123 Perugia, Italy

[2]Institute of Organic Synthesis and Photoreactivity, National Research Council, via P. Gobetti 101, 40129 Bologna, Italy

[3]Consiglio Nazionale delle Ricerche, Istituto per lo Studio dei Materiali Nanostrutturati (CNRISMN), 40129 Bologna, Italy

[4]Laboratorio di Patologia delle Infezioni Associate all'Impianto, IRCCS Istituto Ortopedico Rizzoli, via di Barbiano 1/10, 40136 Bologna, Italy

[5]Department of Experimental, Diagnostic, and Specialty Medicine, University of Bologna, via San Giacomo 14, 40126 Bologna, Italy

[6]Laboratorio di Immunoreumatologia e Rigenerazione Tissutale, IRCCS Istituto Ortopedico Rizzoli, via di Barbiano, 1/10 40136 Bologna, Italy

Corresponding Authors at: Dipartimento di Scienze Farmaceutiche, Università di Perugia, Via del Liceo, 1, 06123 Perugia, Italy. E-mail: morena.nocchetti@unipg.it; anna.donnadio@unipg.it; riccardo.vivani@unipg.it

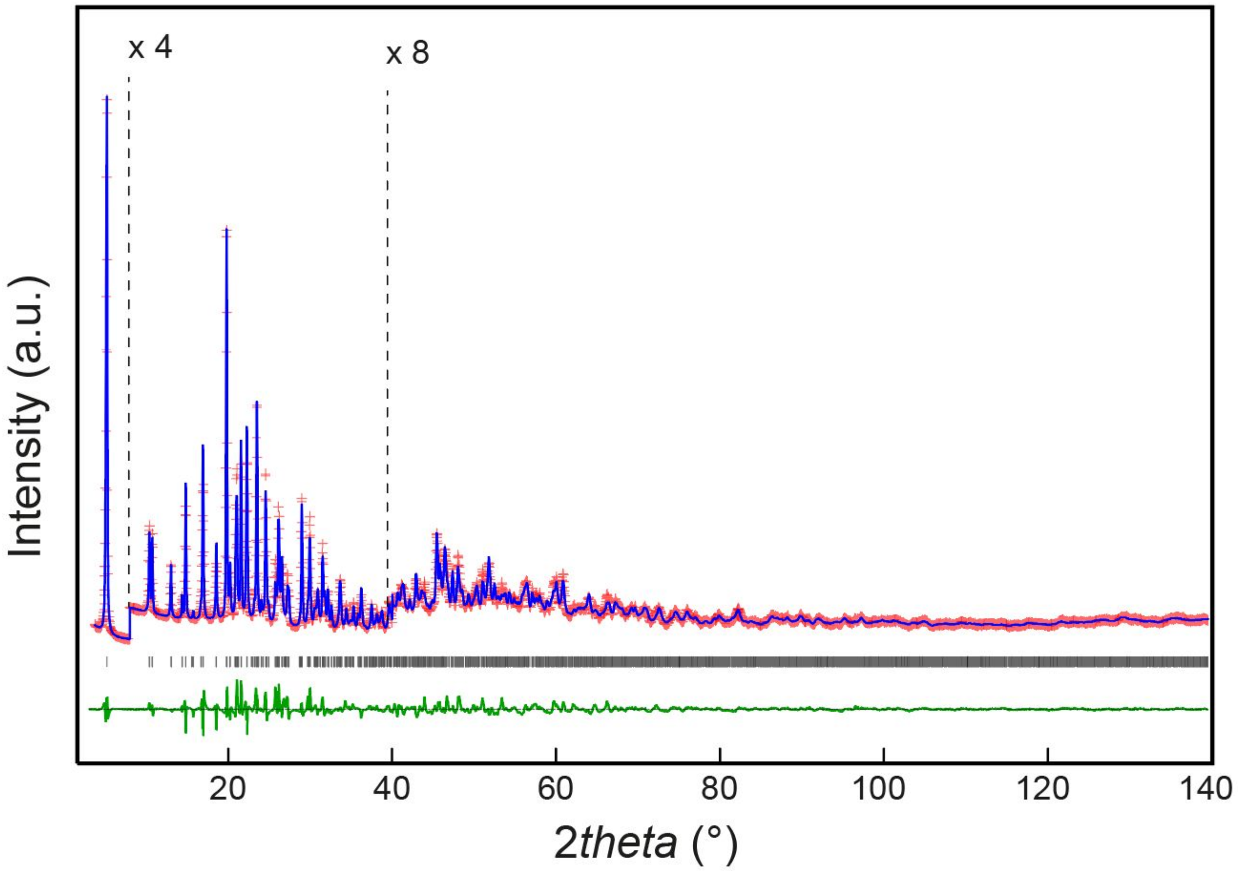

Fig. S1. Rietveld plot of the last refinement cycle for **2**, showing the calculated profile (blue), the experimental pattern (red cross) and their difference (green). Black marks indicate the calculated positions of Bragg peaks.

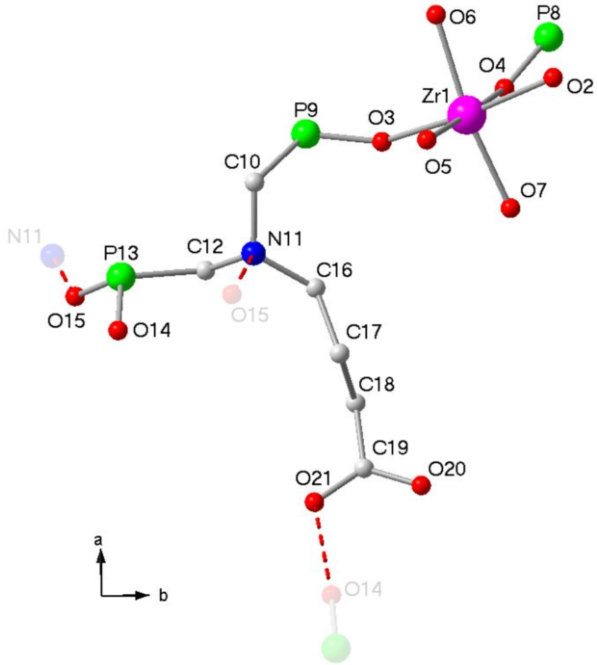

Fig. S2. Asymmetric unit and labelling scheme for **2.**

Table S1: Structural data and refinement details for **2.**

Formula sum	$Zr_2P_5O_{20}N_2C_{12}H_{25}$
Formula weight / g mol^{-1}	854.641
Crystal system	monoclinic
Space-group	*C*2/*c*
a/ Å	34.3460(14)
b/ Å	8.4930(2)
c/ Å	9.0401(2)
α/ deg.	90
β/ deg.	97.15(1)
γ/ deg.	90
Volume/Å^3	2616.54(16)
Z	4
Calculated density/g·cm^{-3}	2.174
Data range/ 2θ·deg^{-1}	3 - 140
Wavelength/Å	1.54059
N. of data points	8059
Reflections collected, unique	2463
N. of parameters	95
N. of restraints	66
R_p	0.045
R_{wp}	0.064
R_F2	0.056
GOF	6.96

$R_p = \Sigma |I_o\text{-}I_c| / \Sigma I_o$; $R_{wp} = [\Sigma w(I_o\text{-}I_c)^2 / \Sigma wI_o^2]^{1/2}$; $R_F2 = \Sigma |F_o^2 - F_c^2| / \Sigma |F_o^2|$;
$GOF = [\Sigma w(I_o\text{-}I_c)^2 / (N_o - N_{var})]^{1/2}$

Table S2: Refined atomic coordinates for **2**

**Atom**	**x/*a***	**y/*b***	**z/*c***
Zr1	0.4544(1)	0.7519(4)	0.43949(31)
O2	0.47585(34)	0.9585(11)	0.3491(12)
O3	0.4392(4)	0.5489(10)	0.5532(14)
O4	0.46993(28)	0.8426(11)	0.6586(10)
O5	0.4400(4)	0.6558(13)	0.2334(10)
O6	0.51413(25)	0.6819(14)	0.4581(15)
O7	0.39880(26)	0.8528(14)	0.4521(15)
P8	0.5	0.9492(11)	0.75
P9	0.44443(23)	0.3679(8)	0.5723(9)
C10	0.4162(4)	0.2441(21)	0.4371(18)
N11	0.3740(4)	0.2394(16)	0.4448(19)
C12	0.3643(6)	0.1242(13)	0.5599(19)
P13	0.36103(22)	-0.0765(9)	0.5029(10)
O14	0.3293(4)	-0.0854(16)	0.3639(15)
O15	0.3498(4)	-0.1867(15)	0.6341(13)
C16	0.3530(5)	0.3862(19)	0.4859(27)
C17	0.3139(4)	0.4417(26)	0.4042(22)
C18	0.2844(4)	0.4781(22)	0.5233(20)

C19	0.24563(29)	0.4961(10)	0.4229(12)
O20	0.2350(4)	0.6316(12)	0.3709(20)
O21	0.2259(4)	0.3769(13)	0.3813(16)

Table S3: Bond lengths for **2**

**Atom 1**	**Atom 2**	**Length/ Å**
Zr1	O2	2.106(7)
Zr1	O3	2.106(8)
Zr1	O4	2.130(8)
Zr1	O5	2.038(8)
Zr1	O6	2.123(8)
Zr1	O7	2.109(8)
P8	O2	1.513(6)
P8	O2	1.513(6)
P8	O4	1.536(7)
P8	O4	1.536(7)
P9	O3	1.555(9)
P9	O5	1.497(9)
P9	O6	1.542(8)
P9	C10	1.801(10)
N11	C10	1.461(11)
N11	C12	1.496(11)
N11	C16	1.510(11)
P13	O7	1.550(8)
P13	C12	1.780(10)
P13	O14	1.559(9)
P13	O15	1.595(9)
C16	C17	1.525(12)
C17	C18	1.596(12)
C18	C19	1.524(12)
C19	O20	1.279(10)
C19	O21	1.249(11)
N11	O15	2.86(2)
O14	O21	2.75(2)

Table S4: Bond angles for **2**

**Atom 1**	**Atom 2**	**Atom 3**	**Amplitude/ deg.**
O2	Zr1	O3	172.3(6)
O2	Zr1	O4	90.0(4)
O2	Zr1	O5	92.0(5)
O2	Zr1	O6	83.3(4)
O2	Zr1	O7	92.6(5)
O3	Zr1	O4	83.7(4)
O3	Zr1	O5	94.1(5)
O3	Zr1	O6	91.5(4)
O3	Zr1	O7	91.5(5)
O4	Zr1	O5	177.6(6)
O4	Zr1	O6	84.3(4)

O4	Zr1	O7	85.8(5)
O5	Zr1	O6	94.7(4)
O5	Zr1	O7	95.4(5)
O6	Zr1	O7	169.2(5)
Zr1	O2	P8	154.8(8)
Zr1	O3	P9	146.3(8)
Zr1	O4	P8	141.9(7)
Zr1	O5	P9	154.3(9)
Zr1	O6	P9	165.3(10)
Zr1	O7	P13	166.0(9)
O2	P8	O2	117.6(11)
O2	P8	O4	111.6(5)
O2	P8	O4	104.0(5)
O2	P8	O4	104.0(5)
O2	P8	O4	111.6(5)
O4	P8	O4	107.7(10)
O3	P9	O5	102.5(7)
O3	P9	O6	110.3(8)
O3	P9	C10	117.2(9)
O5	P9	O6	110.1(8)
O5	P9	C10	118.1(8)
O6	P9	C10	98.6(7)
P9	C10	N11	115.9(10)
C10	N11	C12	111.1(13)
C10	N11	C16	119.8(13)
C12	N11	C16	102.4(12)
N11	C12	P13	115.9(10)
O7	P13	C12	115.6(9)
O7	P13	O14	105.6(8)
O7	P13	O15	106.4(8)
C12	P13	O14	107.0(8)
C12	P13	O15	111.0(8)
O14	P13	O15	111.2(8)
N11	C16	C17	123.7(18)
C16	C17	C18	109.1(12)
C17	C18	C19	101.4(10)
C18	C19	O20	119.91(11)
C18	C19	O21	119.89(10)
O20	C19	O21	119.91(7)

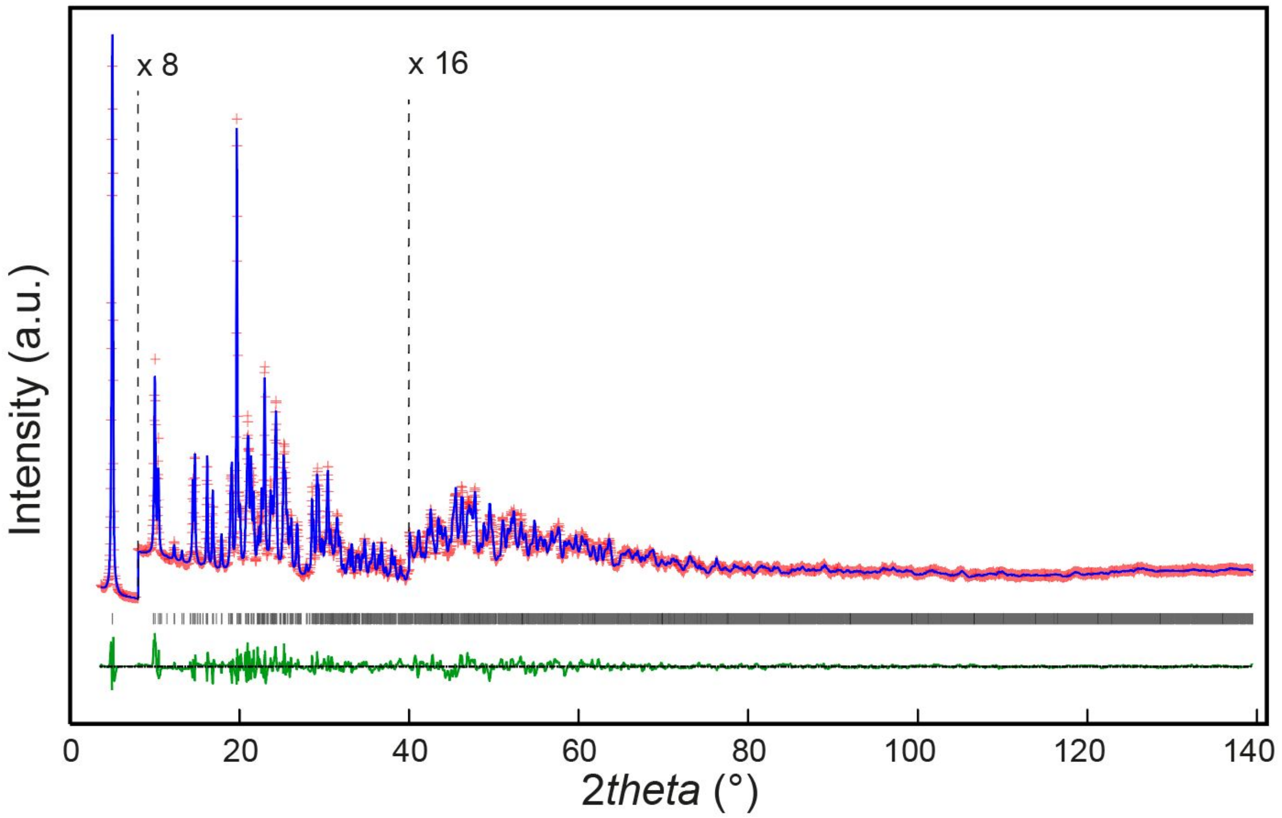

Fig. S3. Rietveld plot of the last refinement cycle for **3**, showing the calculated profile (blue), the experimental pattern (red cross) and their difference (green). Black marks indicate the calculated positions of Bragg peaks.

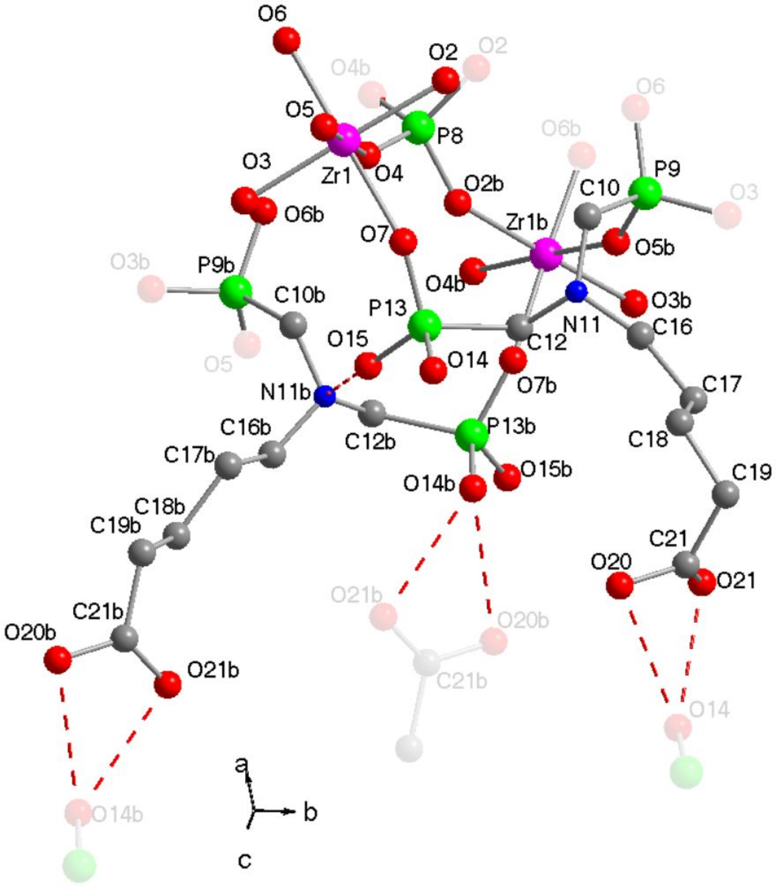

Fig. S4. Asymmetric unit and labelling scheme for **3.**

Table S5: Structural data and refinement details for **3**

Formula	$Zr_2P_5O_{20}N_2C_{14}H_{29}$
Formula weight / g·mol^{-1}	882.83
Crystal system	triclinic
Space-group	*P*-1
a/ Å	17.9803(9)
b/ Å	8.6066(4)
c/ Å	9.04778(29)
α/ deg.	90.4659(32)
β/ deg.	94.910(4)
γ/ deg.	99.552(4)
Volume/ Å^3	1375.30(10)
Z	2
Calculated density/g·cm^{-3}	2.062
Data range/ 2θ·deg^{-1}	3.5 - 140
Wavelength/Å	1.54059
N. of data points	8029
Reflections collected, unique	5184
N. of parameters	185
N. of restraints	140
R_p	0.054
R_{wp}	0.074
R_F2	0.053
GOF	0.80

$R_p = \Sigma |I_o\text{-}I_c| / \Sigma I_o$; $R_{wp} = [\Sigma w(I_o\text{-}I_c)^2 / \Sigma wI_o^2]^{1/2}$; $R_F2 = \Sigma |F_o^2 - F_c^2| / \Sigma |F_o^2|$;
$GOF = [\Sigma w(I_o\text{-}I_c)^2 / (N_o - N_{var})]^{1/2}$

Table S6: Refined atomic coordinates for **3**

**Atom**	**x/*a***	**y/*b***	**z/*c***
Zr1	0.91534(33)	0.2164(7)	0.4511(6)
O3	0.8684(4)	0.0070(7)	0.5396(11)
O5b	0.8841(6)	0.8142(15)	0.7424(9)
O6	0.9830(4)	0.8636(15)	0.5564(14)
O7	0.8115(4)	0.3001(11)	0.4606(12)
P9	0.8985(4)	0.8545(10)	0.5797(10)
C10	0.8438(8)	0.6942(14)	0.4584(17)
N11	0.7689(7)	0.6457(13)	0.4810(15)
C12	0.7557(11)	0.5402(12)	0.5948(16)
P13	0.7405(5)	0.3289(12)	0.5354(11)
O14	0.6681(5)	0.3008(24)	0.4262(17)
O15	0.7293(11)	0.2296(18)	0.6758(13)
C16	0.7220(11)	0.7581(29)	0.470(4)
C17	0.6511(16)	0.837(4)	0.451(4)
C18	0.4040(11)	0.234(4)	0.6812(27)
C19	0.4792(10)	0.1687(30)	0.6832(22)
O20	0.5564(12)	0.4026(19)	0.5853(28)
O21	0.5129(8)	0.1896(32)	0.4243(23)

C21	0.5269(10)	0.2503(21)	0.5626(20)
Zr1b	0.91485(33)	0.7138(7)	0.9426(6)
O3b	0.8928(9)	0.9052(7)	0.0587(14)
O5	0.8786(7)	0.1273(14)	0.2379(7)
O6b	0.9785(5)	0.1705(15)	0.0553(13)
O7b	0.80238(32)	0.6013(12)	0.9443(11)
P9b	0.8976(5)	0.0859(9)	0.0812(9)
C10b	0.8319(8)	0.1570(23)	0.9422(11)
N11b	0.7666(7)	0.2031(14)	0.9808(14)
C12b	0.7753(10)	0.3217(12)	0.0897(14)
P13b	0.7423(5)	0.5067(12)	0.0343(11)
O14b	0.6645(5)	0.4636(21)	0.9409(18)
O15b	0.7319(11)	0.5989(20)	0.1766(13)
C16b	0.7098(13)	0.0763(27)	0.009(4)
C17b	0.6697(21)	0.951(4)	0.8934(28)
C18b	0.6098(16)	0.8318(34)	0.9622(24)
C19b	0.5623(11)	0.7221(34)	0.8404(29)
O20b	0.4546(11)	0.5113(20)	0.8768(27)
O21b	0.4577(12)	0.7583(19)	0.9874(31)
C21b	0.4831(8)	0.6630(21)	0.8932(22)
O2	0.9607(6)	0.4307(12)	0.3613(12)
O2b	0.0601(6)	0.4784(13)	0.1748(13)
O4	0.0505(5)	0.6890(11)	0.3365(8)
O4b	0.9409(5)	0.6039(13)	0.1407(10)
P8	0.0001(5)	0.5491(9)	0.2528(9)

Table S7: Bond lengths for **3**

Atom 1	Atom 2	Length/ Å
Zr1	O3	2.058(7)
Zr1	O6	2.064(6)
Zr1	O7	2.118(6)
Zr1	O5	2.082(6)
Zr1	O2	2.090(8)
Zr1	O4	2.084(6)
P9	O3	1.536(6)
P9	O5b	1.549(6)
P9	O6	1.541(6)
P9	C10	1.848(6)
N11	C10	1.374(6)
N11	C12	1.388(7)
N11	C16	1.385(7)
P13	O7	1.549(6)
P13	C12	1.862(7)
P13	O14	1.548(6)
P13	O15	1.546(6)
C16	C17	1.541(5)
C17	C18	1.537(5)
C18	C19	1.546(5)
C19	C21	1.548(5)

C21	O20	1.337(7)
C21	O21	1.339(7)
Zr1b	O5b	2.085(7)
Zr1b	O3b	2.058(7)
Zr1b	O6b	2.007(7)
Zr1b	O7b	2.093(7)
Zr1b	O2b	2.089(7)
Zr1b	O4b	2.087(7)
P9b	O3b	1.554(6)
P9b	O5	1.539(6)
P9b	O6b	1.553(6)
P9b	C10b	1.830(6)
N11b	C10b	1.371(6)
N11b	C12b	1.393(7)
N11b	C16b	1.407(7)
P13b	O7b	1.537(6)
P13b	C12b	1.848(7)
P13b	O14b	1.558(6)
P13b	O15b	1.548(6)
C16b	C17b	1.538(5)
C17b	C18b	1.538(5)
C18b	C19b	1.545(5)
C19b	C21b	1.548(5)
C21b	O20b	1.324(7)
C21b	O21b	1.337(7)
P8	O2	1.549(6)
P8	O2b	1.541(6)
P8	O4	1.533(7)
P8	O4b	1.541(6)
N11	O15b	2.79(2)
N11b	O15	2.80(2)
O14	O20	2.82(3)
O14	O21	2.79(2)
O14b	O20b	2.85(3)
O14b	O21b	2.79(2)

Table S8: Bond angles for **3**

Atom 1	Atom 2	Atom 3	amplitude/ deg.
O3	Zr1	O6	90.47(32)
O3	Zr1	O7	89.73(33)
O3	Zr1	O5	90.37(30)
O3	Zr1	O2	178.7(4)
O3	Zr1	O4	90.41(29)
O6	Zr1	O7	180.000(4)
O6	Zr1	O5	91.39(30)
O6	Zr1	O2	90.81(33)
O6	Zr1	O4	90.41(28)
O7	Zr1	O5	89.15(31)

O7	Zr1	O2	88.99(34)
O7	Zr1	O4	89.05(27)
O5	Zr1	O2	89.55(30)
O5	Zr1	O4	178.0(4)
O2	Zr1	O4	89.63(31)
Zr1	O3	P9	133.6(7)
P9	O5b	Zr1b	150.2(8)
Zr1	O6	P9	157.1(7)
Zr1	O7	P13	154.6(7)
O3	P9	O5b	109.2(5)
O3	P9	O6	113.1(5)
O3	P9	C10	107.7(5)
O5b	P9	O6	110.6(5)
O5b	P9	C10	108.0(5)
O6	P9	C10	108.1(5)
P9	C10	N11	117.9(6)
C10	N11	C12	115.4(7)
C10	N11	C16	117.2(7)
C12	N11	C16	114.7(7)
N11	C12	P13	114.6(7)
O7	P13	C12	107.6(5)
O7	P13	O14	112.2(6)
O7	P13	O15	111.7(6)
C12	P13	O14	106.6(5)
C12	P13	O15	107.4(5)
O14	P13	O15	111.1(6)
N11	C16	C17	162.2(22)
C16	C17	C18	112.3(6)
C17	C18	C19	110.7(6)
C18	C19	C21	109.3(6)
C19	C21	O20	117.3(7)
C19	C21	O21	117.5(7)
O20	C21	O21	120.2(8)
O5b	Zr1b	O3b	90.58(29)
O5b	Zr1b	O6b	91.45(32)
O5b	Zr1b	O7b	88.29(31)
O5b	Zr1b	O2b	89.58(28)
O5b	Zr1b	O4b	177.0(4)
O3b	Zr1b	O6b	86.4(5)
O3b	Zr1b	O7b	91.3(4)
O3b	Zr1b	O2b	178.7(5)
O3b	Zr1b	O4b	90.67(29)
O6b	Zr1b	O7b	177.6(5)
O6b	Zr1b	O2b	92.3(4)
O6b	Zr1b	O4b	91.30(32)
O7b	Zr1b	O2b	90.02(33)
O7b	Zr1b	O4b	89.00(31)
O2b	Zr1b	O4b	89.23(30)
Zr1b	O3b	P9b	151.3(8)
Zr1	O5	P9b	149.1(7)

Zr1b	O6b	P9b	170.7(8)
Zr1b	O7b	P13b	146.6(6)
O3b	P9b	O5	111.4(5)
O3b	P9b	O6b	109.4(5)
O3b	P9b	C10b	109.1(5)
O5	P9b	O6b	109.7(5)
O5	P9b	C10b	109.8(5)
O6b	P9b	C10b	107.2(5)
P9b	C10b	N11b	121.2(6)
C10b	N11b	C12b	116.2(7)
C10b	N11b	C16b	113.5(7)
C12b	N11b	C16b	111.9(7)
N11b	C12b	P13b	116.4(7)
O7b	P13b	C12b	108.0(5)
O7b	P13b	O14b	111.7(6)
O7b	P13b	O15b	111.1(6)
C12b	P13b	O14b	108.3(5)
C12b	P13b	O15b	108.3(5)
O14b	P13b	O15b	109.3(6)
N11b	C16b	C17b	125.2(17)
C16b	C17b	C18b	111.2(6)
C17b	C18b	C19b	110.4(6)
C18b	C19b	C21b	108.9(6)
C19b	C21b	O20b	118.5(7)
C19b	C21b	O21b	116.4(6)
O20b	C21b	O21b	122.5(8)
Zr1	O2	P8	159.9(8)
Zr1b	O2b	P8	146.8(8)
Zr1	O4	P8	142.5(7)
Zr1b	O4b	P8	149.7(7)
O2	P8	O2b	111.1(6)
O2	P8	O4	111.3(5)
O2	P8	O4b	110.4(5)
O2b	P8	O4	100.2(7)
O2b	P8	O4b	111.9(5)
O4	P8	O4b	111.5(6)

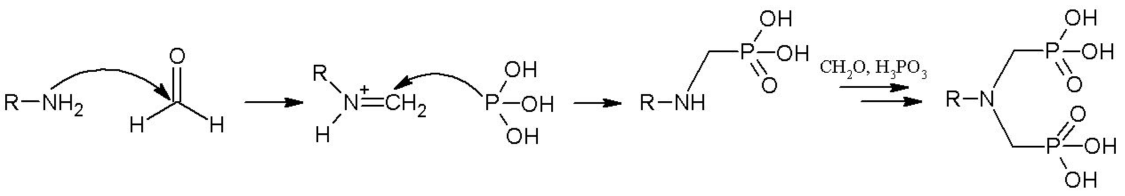

Scheme S1. Moedritzer- Irani reaction[1].

Thermal behavior of ZP

Fig. S5 shows the TGA curves for ZP. TGA curve for **1** was reported in ref. 2. As can be observed, the thermal profiles are quite similar. It can be noted from all graphs, an initial small weight loss at ca. 150 °C which can likely be attributed to the loss of adsorbed water (0.6, 0.7, 1 mol per formula unit for **2**, **3** and **4** respectively). The second step of weight loss (ca.11%), occurring between 150 and 330 °C can be ascribed to the decarboxylation of the organic moieties which corresponds to the loss of two CO_2 mol per formula unit. The following steps are not resolved, but they can be attributed to the combustion of the organic part to yield ZrP_2O_7 and P_2O_5. Finally, the solids recovered from the TGA were characterized by XRPD, and only cubic ZrP_2O_7 was detected. The total weight loss at 1200 °C is 30%, 32%, 36% for **2**, **3** and **4** respectively. The experimental and the calculated formula weights, compared in Table S9, are in good agreement with an error of 1.5% at most.

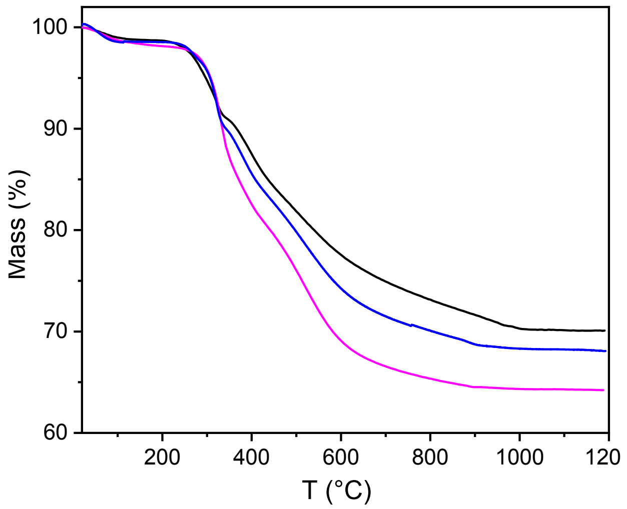

Fig. S5. TGA curves of: **2** (black), **3** (blue) and **4** (magenta). Operative conditions: air flow; heating rate: 10°C/min.

Table S9. Experimental formula weights of ZP having general formula $Zr_2(PO_4)\ H_5(L)_2{\cdot}mH_2O$ determined from TGA. For comparison calculated formula weights (FW) were reported.

Sample	H_2O (mol/formula unit)	FW_{calc} (anhydrous)	FW_{exp} (anhydrous)
**2**	0.6	854	852
**3**	0.7	882	874
**4**	1	910	924

Study on the ZP reactivity

The ion exchange properties of solids were investigated by titrating **ZP** microcrystals with 0.1 M KOH (Fig. S6 (a)), 0.1 M $MeNH_2$ and 0.1 M $PrNH_2$ in the presence of KCl, $PrNH_3^+Cl^-$ and $MeNH_3^+Cl^-$ salts respectively (Fig. S7).

In general, the titration curves of ZP with KOH show the ability of the solids to exchange hydrogens with K^+ cations. The presence of KCl in the solid suspensions provokes the exchange of the protons on the surface by K^+ ions lowering the pH of the suspensions at 3.3 for **1** and **3**, and 3.6 and 3.8 for **4** and **2** respectively. Firstly, the exchanged protons are titrated by KOH causing an abrupt improvement of the pH. After that, in **1** and **2**, the pH remained almost constant in response to a further addition of KOH suggesting that the H^+/K^+ exchange involves the interlayer region with the formation of intermediate phases[3]. Endpoints were observed from which the experimental ion exchange capacity (IEC) of 3.6 and 3.9 mequiv/g was obtained for **1** and **2**, respectively. In contrast to the behaviour of **1** and **2**, in **3** and **4** each increment of metal ion uptake resulted in an increase in pH and no definite plateaux as well as sharp end points were observed. This can be explained considering that **3** and **4**, after a certain exchange degree, undergo a progressive exfoliation and the exchange occurs for the most part on the surfaces. In this case it was not possible to determine exactly the experimental IEC, thus it was estimated at pH equal to 10 and resulted to be 3.5 and 3.0 mequiv/g for **3** and **4**, respectively.

The experimental IEC is close to those calculated considering three exchangeable hydrogens per mol of Zr phosphonate (three protons bonded on one POH and two COOH, the protons bonded to N are not enough acid to be exchanged). As an example, the IEC calculated for **1** was:

$$\left(\frac{1000}{798\frac{g}{mol}}\right) \times 3 = \ 3.8\, meq/g$$

where 798 is the anhydrous molecular weight of **1**. The calculated IEC for **2**, **3** and **4** is 3.5, 3.4, 3.3 mequiv/g, respectively.

The interlayer distances (*d*) of dried solids recovered at the end of the titration increase linearly as the length of the alkyl chain increases (Fig. S4 (b)) according to the presence of the metal ions in the galleries even if the increase of *d* does not correspond to the ion diameter. This can be explained considering that the K^+ cations can be accommodated in the cavity formed in the interlayer regions.

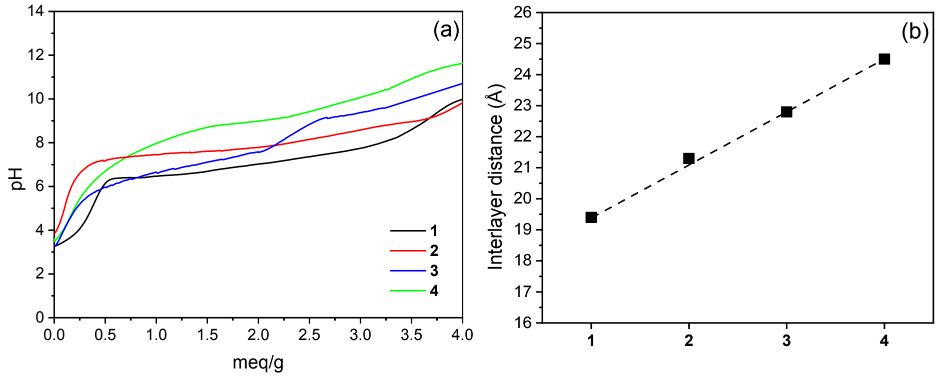

Fig. S6. Titration curves with KOH 0.1 M in the presence of KCl 1M (a); interlayer distances of dried solids after titration with KOH (b).

The titration curves with propylamine and methylamine were reported in Fig. S7. The sample **1** shows a behaviour different from the other ZP; after a first burst increase of pH, the pH of the dispersion of **1** remains almost constant as the amine reached the 50% (1.8 mequiv/g) and the 33 % (1.2 mequiv/g) of the exchange for methylamine and propylamine, respectively. Until these percentages of exchange, the formation of two phases and no extended exfoliation are expected. Additional amines were exchanged with a progressive pH increase as the exchange takes place on the surface of independent exfoliated lamellae. This behaviour is typical of exfoliated 2D materials; indeed, it was observed that once the added amine reached at least 60% of the IEC a colloidal dispersion was obtained. Samples **2**, **3** and **4** did not show any plateau, after a first sudden pH increase, the pH increases more or less rapidly. In these samples a certain degree of exfoliation is expected for low amine exchange.

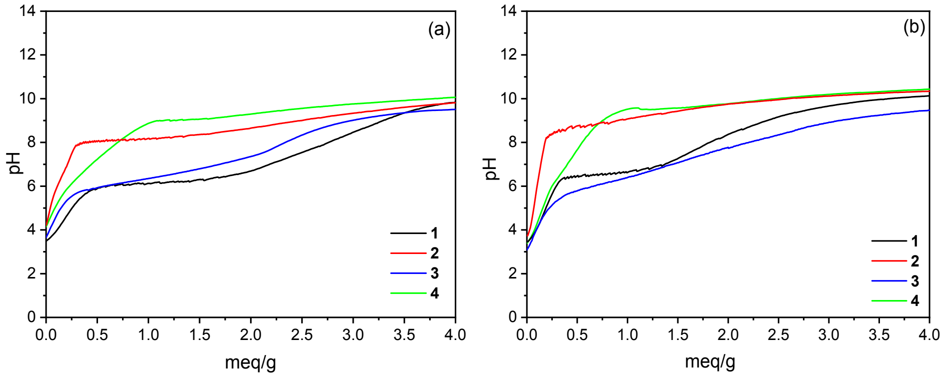

Fig. S7. Titrations with 0.1 M of (a) $MeNH_2$, (b) $PrNH_2$ in the presence of $PrNH_3^+Cl^-$ and $MeNH_3^+Cl^-$ 1M.

Characterization of colloidal dispersions of ZP

Table S10. Zr, P, C, N contents (mol/FW) in the regenerated solids.

Sample	Zr exp	Zr calc	P exp	P calc	C exp	C calc	N exp	N calc
**1**	2	2	4.8	5	9.2	8	2.26	2
**2**	2	2	4.8	5	18.1	12	2.28	2
**3**	2	2	4.9	5	15.3	14	2.14	2
**4**	2	2	4.9	5	12.1	16	1.8	2

Antibacterial properties of Ag@ZP

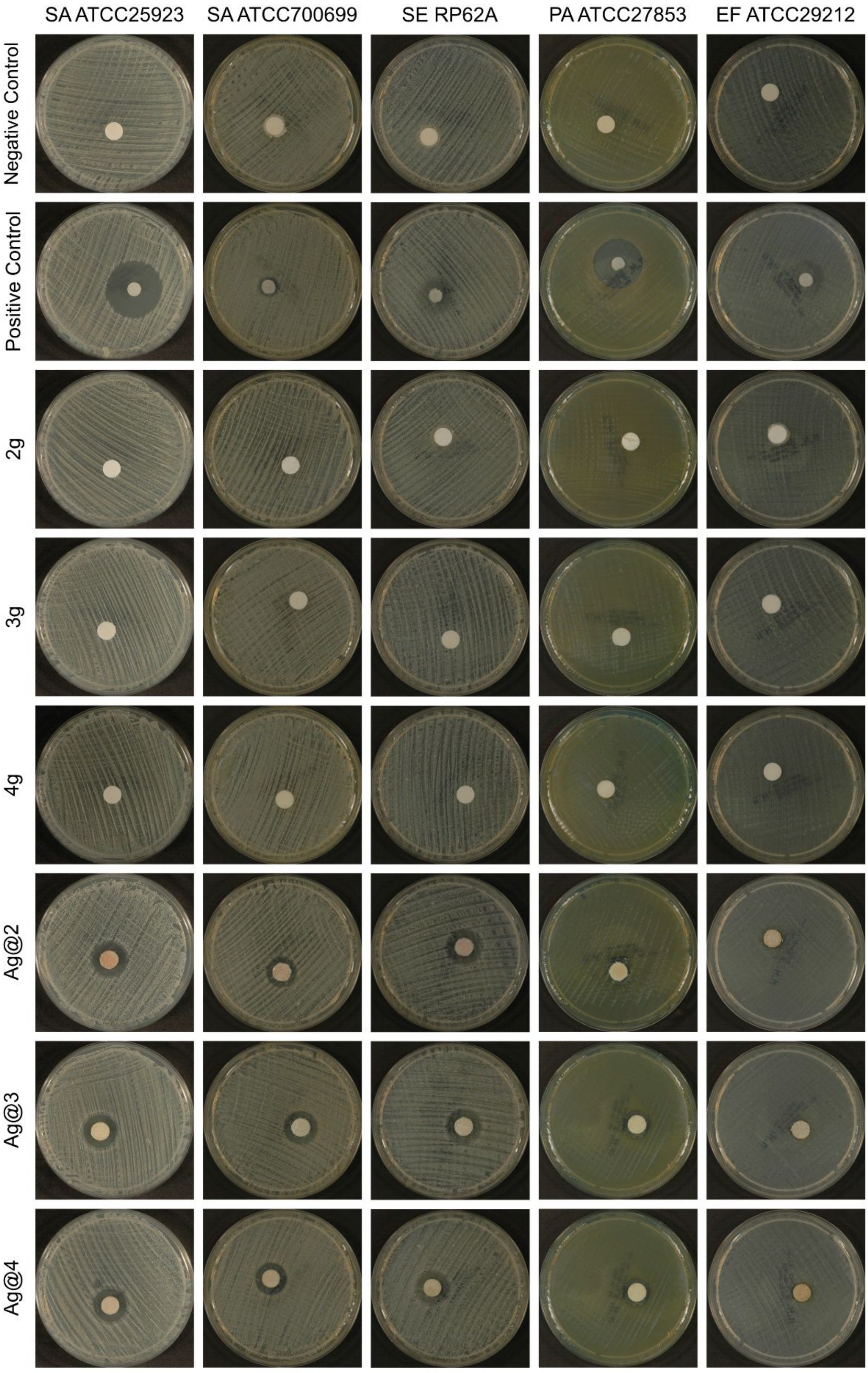

Fig. S8. Representative plates of three independent experiments of agar diffusion assay are shown. The photographs of the positive control refer to plates treated with the gentamicin antibiotic. SA, *S. aureus*; SE, *S. epidermidis*; PA, *P. aeruginosa*; EF, *E. faecalis*.

Table S11. Zones of inhibition (mm) of different Ag@ZP on *S. epidermidis* and *E. faecalis.* Legend: GEN, positive control consisting of gentamicin (50 μg); P/S, positive control consisting of penicillin (50 U) and streptomycin (0.05 mg).

TREATMENT	*Staphylococcus epidermidis* ATCC RP62A		*Enterococcus faecalis* ATCC 29212	
	Mean	S.D.	Mean	S.D.
Negative Control	0.0	0.0	0.0	0.0
2g	0.0	0.0	0.0	0.0
3g	0.0	0.0	0.0	0.0
4g	0.0	0.0	0.0	0.0
Ag@2	5.0	0.5	1.0	0.0
Ag@3	5.2	0.8	1.2	0.3
Ag@4	5.0	0.0	1.0	0.0
Positive Control (GEN)	4.9	1.0	6.5	0.3
Positive Control (P/S)	5.3	2.8	9.8	0.3

Table S12. Zones of inhibition (mm) of different Ag@ZP on *S. aureus.* Legend: GEN, positive control consisting of gentamicin (50 μg); P/S, positive control consisting of penicillin (50 U) and streptomycin (0.05 mg).

TREATMENT	*Staphylococcus aureus* ATCC 25923 (MSSA)		*Staphylococcus aureus* ATCC 700699 (MRSA/VISA)	
	Mean	S.D.	Mean	S.D.
Negative Control	0.0	0.0	0.0	0.0
2g	0.0	0.0	0.0	0.0
3g	0.0	0.0	0.0	0.0
4g	0.0	0.0	0.0	0.0
Ag@2	3.8	0.8	3.7	0.3
Ag@3	3.7	0.3	4.3	0.3

Ag@4	4.0	0.0	4.0	0.5
Positive Control (GEN)	11.8	0.3	2.2	0.4
Positive Control (P/S)	20.9	0.4	11.7	0.8

Table S13. Zones of inhibition (mm) of different Ag@ZP on *P. aeruginosa.* Legend: GEN, positive control consisting of gentamicin (50 μg); P/S, positive control consisting of penicillin (50 U) and streptomycin (0.05 mg).

TREATMENT	*Pseudomonas aeruginosa* ATCC 27853	
	Mean	S.D.
Negative Control	0.0	0.0
2g	0.0	0.0
3g	0.0	0.0
4g	0.0	0.0
Ag@2	2.3	0.3
Ag@3	2.7	0.3
Ag@4	2.8	0.6
Positive Control (GEN)	10.6	0.2
Positive Control (P/S)	7.6	0.7

References

(1) Moedritzer K.; Irani R. R. The Direct Synthesis of α-Aminomethylphosphonic Acids. Mannich-Type Reactions with Orthophosphorous Acid. *J. Org. Chem.*, **1966**, *31*, 1603−1607.

(2) Donnadio A.; Nocchetti M.; Costantino F.; Taddei M.; Casciola M.; da Silva Lisboa F.; Vivani R. A Layered Mixed Zirconium Phosphate/Phosphonate with Exposed Carboxylic and Phosphonic Groups: X-ray Powder Structure and Proton Conductivity Properties, *Inorg. Chem.*, **2014,** *53,* 13220-13226.

(3) Alberti A.; Costantino U. in Solid-State Supramolecular Chemistry: Two and Three-dimensional Inorganic Networks, Vol. 7 of Comprehensive Supramolecular Chemistry, G. Alberti and T. Bein Vol Eds., (Pergamon, Elsevier Science Ltd Press, 1996) p. 1.
